# Knowledge-guided graph fusion of mRNA profiles for interpretable cancer subtyping

**DOI:** 10.1093/bfgp/elag009

**Published:** 2026-10-01

**Authors:** Jie Ni, Xinting Zhang, Mingyang Li, Yun Liu, Adam Jatowt

**Affiliations:** School of Biological Science and Medical Engineering, Southeast University, No. 2 Southeast University Road, Jiangning District, Nanjing, Jiangsu 211102, China; Department of Medical Informatics, School of Biomedical Engineering and Informatics, Nanjing Medical University, 101 Longmian Avenue, Jiangning District, Nanjing, Jiangsu 211166, China; Data Science Group, Digital Science Center, University of Innsbruck, Innrain 15, 6020 Innsbruck, Tirol, Austria; School of Biological Science and Medical Engineering, Southeast University, No. 2 Southeast University Road, Jiangning District, Nanjing, Jiangsu 211102, China; School of Biological Science and Medical Engineering, Southeast University, No. 2 Southeast University Road, Jiangning District, Nanjing, Jiangsu 211102, China; School of Biological Science and Medical Engineering, Southeast University, No. 2 Southeast University Road, Jiangning District, Nanjing, Jiangsu 211102, China; Department of Information, the First Affiliated Hospital, Nanjing Medical University, 300 Guangzhou Road, Gulou District, Nanjing, Jiangsu 210029, China; Data Science Group, Digital Science Center, University of Innsbruck, Innrain 15, 6020 Innsbruck, Tirol, Austria

**Keywords:** knowledge-guided learning, graph convolutional networks, single-transcriptome classification, historical candidate challenge, auditable machine learning

## Abstract

Accurate cancer subtype classification from single-transcriptome data remains challenging because of biological heterogeneity, measurement noise, and limited sample sizes. This study examined whether disease–gene priors add predictive information under strict separation of association-edge, patient-sample, and external-transfer boundaries. It also audited a historically selected 12-gene subset without claiming de novo discovery. Important mRNA Identification through Hybrid Fusion (ImRIHF) uses a layer attention graph convolutional network to learn cancer-specific association scores. A graph convolutional network for disease classification (GCNDC) then classifies subtypes after score-guided expression weighting. Association folds were masked before both Gaussian kernels and the heterogeneous graph were rebuilt. Patient preprocessing, feature selection, graph construction, tuning, and fitting remained training-contained. Twenty repeated five-fold refits described stability, while separate locked audits supplied paired effects. Patient-disjoint external transfer used frozen development predictors and no target-cohort fitting. Matched GCNDC and full-transcriptome controls bounded interpretation within one fixed 29-coordinate multiplicity family. UCEC remained feasibility-only because its locked audit contained 10 MSEAC cases. Leakage-controlled association AUPR and AUROC were 0.932 and 0.941, respectively. Non-UCEC locked effects versus matched GCNDC ranged from *+0.027* to *+0.035*; the UCEC effect was *+0.032* and remained descriptive. Strict-common external effects were *+0.034* in METABRIC, *+0.026* in CGGA, and *+0.036* in GSE14333. Of 12 historical challenge genes, 11 mapped, 4 were BH-significant, and 2 were both direction-concordant and BH-significant. The frozen disease-guided rule showed modest positive differences from matched controls in the named retrospective cohorts. Full-transcriptome controls, UCEC feasibility limits, and source-specific transfer boundaries restrict this result. The analyses do not establish generic superiority, prospective validity, clinical utility, population generalization, causality, or a validated biomarker panel.

## Introduction

Cancer-subtype classification seeks to resolve molecularly distinct disease groups. mRNA expression offers a comparatively accessible view of gene activity and can contain subtype information, but heterogeneity, high dimensionality, noise, and limited sample size can impair classification from raw profiles [1–9]. We therefore evaluate a computational classification method; transcriptome-only prediction is not presented as evidence of diagnosis, treatment benefit, or a biological mechanism. Multi-omics integration can provide complementary information, but it also adds assay, harmonization, and missing-data burdens [10–12]. Improving a classifier that uses one patient mRNA assay is, therefore, useful when only transcriptomic data are available. This motivation does not imply that single-transcriptome prediction outperforms multi-omics integration or is economically cost-effective. The emergence of the theory-guided data science (TGDS) paradigm has been driven by the lack of interpretability in black-box data science models such as deep learning. TGDS not only learns patterns automatically from training data but also values the treasure trove of accumulated scientific knowledge, showing promise in addressing scientific problems across various disciplines. TGDS posits that using prior knowledge to guide data science can significantly enhance model performance [13]. We used RNADisease v4.0 as the source of curated RNA-disease associations [14]. We treat RNADisease as an incomplete, historically accumulated guidance prior rather than verified ground truth. DisGeNET and Open Targets provide source-reconstruction sensitivity, while an end-to-end residual graph convolutional network (ERGCN) and a graph convolutional network-based approach for clustering and classification (GCNCC) exemplify alternative graph-based classifiers; none is evidence that the proposed hybrid fusion must succeed [15–18]. This study therefore tests disease-conditioned weighting against explicit comparators and failure gates rather than assuming that intermediate fusion improves performance. To explore the potential of intermediate fusion in cancer subtype classification mediated by mRNA expression data, we developed a computational framework called Important mRNA Identification through Hybrid Fusion (ImRIHF), a hybrid-fusion framework built on two graph-convolutional modules: an LAGCN for early fusion and a GCN for disease classification (GCNDC) for intermediate fusion and classification. The analysis treats the frozen mRNA–disease association matrix as a candidate guidance source rather than reliable ground truth. LAGCN was evaluated at an association-edge boundary, whereas GCNDC and the composed ImRIHF pipeline were evaluated at a patient-sample boundary. The resulting frozen weighted rule was assessed against its prespecified comparators for paired F1 or macro-F1 differences, suppression of uninformative variation, and reproducibility of candidate rankings. The paired estimand is conditional on the training data, fitted models, class set, and prediction rule and does not include retraining uncertainty or establish generic method superiority. The leakage-contained results and case studies document retrospective performance but do not establish *de novo* biomarker discovery, a general biological mechanism, or clinical utility.

Accurate subtype classification from one mRNA assay can be limited by noise, heterogeneity, and sample scarcity. ImRIHF combines a graph-derived cancer–mRNA prior with patient expression and evaluates this cross-task transfer hypothesis through leakage-contained nested evaluation, full-transcriptome controls, prior and graph nulls, locked retrospective transfer, and explicit failure gates. The study reports retrospective performance differences but does not claim validated biomarkers, monetary cost-effectiveness, clinical decision support, or a general biological mechanism. ImRIHF combines a layer attention graph convolutional network (LAGCN) for early fusion with a graph convolutional network for disease classification (GCNDC) for association-guided expression weighting and subtype prediction. We tested whether gene-to-prior alignment, rather than generic rescaling or damping, explains a positive paired F1 or macro-F1 difference between the frozen disease-guided and comparator prediction rules under leakage-controlled association learning, fully nested patient evaluation, source reconstruction, graph nulls, and locked transfer. Prior work uses biological networks to regularize expression models, construct patient graphs, integrate multiple omics, or infer subtype-specific interaction networks. Relation-network graph convolution, a graph-embedded deep feedforward network (GEDFN), Multi-Omics Graph cOnvolutional NETworks (MOGONET), ERGCN, GCNCC, and GeSubNet exemplify these strategies [11, 17–21]. GeSubNet jointly learns subtype structure and gene interactions from patient expression, whereas ImRIHF asks a distinct transfer question: can a historically assembled cancer–gene association model, filtered without using evaluated-patient profiles or labels, provide useful geometry for subtype classification? Its scores enter as a cancer-conditioned diagonal metric, rather than as patient labels or an additional omics modality. The methodological contribution is an auditable cross-task knowledge-transfer estimand tested against prespecified unweighted and full-transcriptome comparators, with score-permuted, matched-marginal, wrong-disease, curation-intensity, alternative-prior, graph-null, and graph-construction analyses used only as robustness vetoes. General reproducibility practices are established and are not claimed as novel here; the narrower contribution is the biology-specific separation of association-edge, patient-sample, candidate-selection, and transfer boundaries. We do not claim novelty for LAGCN, GCNDC, graph convolution, diagonal rescaling, or generic reproducibility infrastructure in isolation. Unlike patient-graph classifiers and network-constrained expression models, ImRIHF estimates a cancer-conditioned disease–gene prior at a separate association-edge boundary and freezes it before patient-model fitting; unlike GeSubNet, its evaluated endpoint is patient-level subtype prediction rather than subtype-specific interaction recovery. The contribution is therefore the falsifiable cross-task transfer estimand and its boundary-separated evaluation, not a new generic GNN layer or a superiority claim over native subtype-network-generation methods. We treat this database as an incomplete, historically accumulated, and potentially noisy prior rather than ground truth. The headline prior excludes expression-only, prognostic, subtype-outcome, evaluated-cohort, and overlapping evidence-source records. Its value must therefore be tested against score permutation, degree-preserving graph nulls, controlled edge addition and deletion, the broader unfiltered RNADisease graph, and a source-disjoint reconstruction from DisGeNET and Open Targets. No individual-level expression profile or subtype label from an evaluated patient is used to construct or tune the prior. Nevertheless, curated evidence may reflect population-level expression studies, disease ascertainment, publication intensity, and other historically accumulated information. We therefore describe the object as a cohort- and evidence-source-disjoint non-expression prior, not as statistically label-independent. ImRIHF tests a falsifiable enrichment hypothesis: genes assigned greater relevance to the parent cancer carry, on average, more subtype-discriminative signal relative to within-subtype variability. The filtered prior uses no evaluated-patient profile or label, but it remains historically informed and cannot establish outcome ignorance. It changes the geometry used to construct the patient graph. The evaluation isolates gene-to-prior alignment from generic rescaling, magnitude damping, graph density, and shared evidence. Any retrospective conclusion requires direct patient-paired method comparisons, concordant source and transform robustness, locked external transfer, and claim-specific failure rules.

## Materials and methods

### Known mRNA–disease association

RNADisease v4.0 provided the disease–gene association source [14]. The archived RNADisease v4.0 ‘Experimental Data’ category contained 20 280 associations between 12 768 mRNAs and 406 diseases. The historical preprocessing intersected those records with the available expression matrices, producing 9182 associations between 4928 mRNAs and 406 diseases. These archived counts document the submitted analysis but do not define the leakage-controlled gene universe used for the completed evaluation.

Let $A\in \{0,1\}^{p\times d}$ denote the binary association matrix, with mRNAs in rows and diseases in columns:


\begin{align*} & A_{ic}=\begin{cases} 1, & \text{if mRNA} i\text{ is associated with disease} c,\\ 0, & \mathrm{otherwise.} \end{cases} \end{align*}

The completed analysis rebuilt this object from the complete eligible precutoff RNADisease record. Every mRNA identifier was mapped independently of archived expression-panel membership. At each patient-fitting boundary, the usable association vector was intersected with the technical-pass gene universe defined from the current training patients. No held-out expression value, held-out subtype label, archived 2000-gene panel, or archived 4928-gene membership defined that universe. The source-first reconstruction and its failure rules are detailed in Supplementary Method S1 under ‘Association-edge containment’.

The primary temporal boundary was 31 December 2022. Records first supported after that date were excluded from every training input and used only for a temporally separated enrichment analysis. The headline prior also excluded expression-only, prognostic, subtype-outcome, evaluated-cohort, and traceably overlapping evidence records. The disease node for each cohort was selected by one exact preferred-label rule fixed before patient analysis. The complete normalization and eligibility rules appear in Supplementary Method S1 and Table S1A.

The required parent-cancer labels were ‘colorectal cancer’ for COADREAD, ‘endometrial cancer’ for UCEC, ‘soft tissue sarcoma’ for SARC, ‘breast cancer’ for BRCA, and ‘glioma’ for LGG. Matching used normalized exact equality to one preferred-label record. Zero or multiple matches made the cohort ineligible. Synonym expansion, ontology descent, nearest-term substitution, and post-result aggregation were not permitted.

A source-reconstruction sensitivity used frozen DisGeNET and Open Targets inputs [15, 16]. Traceable publications, accessions, and evidence sources shared with RNADisease or temporal validation were removed before mapping. This analysis tested robustness to the prior source but was not treated as statistically independent replication. Five prespecified curation controls represented gene degree, evidence-record count, publication count, a matched wrong-disease prior, and technical measurability. Their exact construction, the alternative-prior audit, and the K01–K05 contrasts are reported in Supplementary Method S1 and Tables S1E and S4D.

For association evaluation, positive edges were divided into five disjoint folds. In each outer fold, held-out positives were set to zero before either Gaussian interaction-profile kernel or the heterogeneous graph was constructed. The graph and LAGCN were then refitted without access to those edges. Training and test unknowns were sampled separately and deduplicated across roles. Hyperparameters were selected by inner edge-level cross-validation using mean AUPR, with AUROC as the prespecified tie-break. This association-edge boundary is distinct from every patient-sample boundary below.

The cutoff, cohort roles, endpoints, and analysis rules were fixed before the completed outputs were inspected. However, the public cohorts, their labels, and earlier analyses were historically known. We, therefore, describe the evaluations as locked retrospective analyses rather than outcome-naive confirmation. Supplementary Method S1 records the execution lock, prior-exposure disclosure, and evidence roles.

### mRNA expression datasets

The internal analyses used release-level unselected mRNA expression for five TCGA-derived tasks [5]. COADREAD distinguishes colon from rectal primary tumours. UCEC distinguishes endometrioid endometrial adenocarcinoma (EEA), mixed serous and endometrioid adenocarcinoma (MSEAC), and serous endometrial adenocarcinoma (SEA). SARC distinguishes dedifferentiated liposarcoma (DDLPS), leiomyosarcoma (LMS), and a frozen pooled-other class. BRCA reconstructs the four core PAM50 classes after excluding Normal-like cases and PAM50-defining predictors. Because PAM50 labels are expression-derived, BRCA remains an expression-label benchmark. LGG uses DNA-defined IDH and 1p/19q classes, and it excludes label-defining genes from candidate evidence (Table 1).

**Table 1:** Internal cohorts, endpoints, sample counts, and evidence roles

Cohort	Endpoint labels	Total *n*	Evidence role
COADREAD	COAD/READ	332	Anatomical-site benchmark
UCEC	EEA/MSEAC/SEA	430	Histopathological feasibility analysis
SARC	DDLPS/LMS/pooled other	257	Histopathological subtype audit
BRCA	Basal/HER2/Luminal A/Luminal B	1036	Expression-derived PAM50 benchmark
LGG	IDH-wildtype/non-codeleted/codeleted	511	Label-independent molecular endpoint

Release reconstruction retained 332 COADREAD patients (254 COAD and 78 READ) and 430 UCEC patients (311 EEA, 21 MSEAC, and 98 SEA). It also retained 257 SARC patients (58 DDLPS, 103 LMS, and 96 pooled-other cases). The BRCA and LGG manifests contained 1036 and 511 patients. Supplementary Table S1C provides the release mapping, duplicate resolution, exclusions, and final class counts for every cohort. The archived 2000-gene panels were retained only as historical provenance. They supplied no feature list, preprocessing statistic, model weight, candidate rank, or inferential result to the completed analysis.

Patient-disjoint external analyses used METABRIC for BRCA, CGGA for LGG, and GSE14333 for COADREAD. The eligible denominators were 1559, 286, and 289 patients, respectively. GSE14333 comprised 250 colon and 39 rectal tumours after one record with a missing location was excluded. GSE21050 was retained as a descriptive SARC sensitivity because its pooled undifferentiated category was only partially lineage matched. GSE17025 lacked MSEAC and could not evaluate the primary three-class UCEC endpoint. External cohort composition and endpoint mapping are reported in Supplementary Method S1 and Tables S3A–S3C.

All patient-expression operations were contained within the applicable training boundary. Raw counts received sample-wise $ \log _{2}(\mathrm{CPM}+1)$, other non-negative abundance values received $\log _{2}(x+1)$, and documented log-scale releases retained their declared scale. Genes failed technical filtering when training missingness exceeded 5%, detection failed the declared CPM rule, or the post-transform training interquartile range was zero. Each sample was midrank-normal scored over all observed genes in the current technical-pass universe. Training-gene means and standard deviations were then applied to held-out samples. A missing held-out selected feature received the training mean and therefore became standardized zero.

Feature ranking also remained within training data. Methods without a native selector used the multiclass one-way ANOVA *F* statistic. Undefined values ranked last, and version-stripped Ensembl identifiers resolved exact ties. The candidate feature counts were 64, 128, 256, and 512. FullTx-EN was the prespecified exception and used every technically retained gene under elastic-net regularization. The exact input-scale, filtering, ranking, tie, and missing-feature rules appear under ‘Nested patient-sample evaluation’ in Supplementary Method S1.

The association-eligible manifest was formed only after technical filtering and normal-score transformation. ImRIHF, matched GCNDC, and other information-matched comparators, therefore, received the same candidate genes at each fitting boundary. A method-native graph could cover a declared subset under its frozen missing-gene rule, but it could not introduce another candidate gene. Native, annotation-portability, and strict-common scopes remained separately labelled and were never substituted after results were known.

Two full-transcriptome controls separated association-guided modelling from unrestricted transcriptome prediction. FullTx-EN used multinomial elastic-net regression, and FullTx-GCNDC used the unweighted graph-classification pipeline. Neither received RNADisease, an association-derived filter, an association score, Equation (7), or an archived panel. Information-matched methods instead shared the training-boundary association-eligible manifest. Every method retained its declared native information set, while the strict-common graph analysis was restricted to ImRIHF and matched GCNDC. Supplementary Tables S1B, S4B, and S8 document comparator coverage and finite search budgets.

Cross-platform transfer used a frozen single-sample procedure rather than target-cohort batch correction. Versioned platform annotations mapped probes to version-stripped Ensembl genes. Probes mapping to multiple genes were discarded, and repeated valid probes were collapsed by within-sample median. Annotation availability fixed the anchor universe before target expression or labels were inspected. Rank transformation and standardization used development-frozen rules, and each external patient connected only to frozen development anchors. No target-to-target edge or target-derived location, scale, quantile, batch, label, or outcome parameter was permitted. Supplementary Method S1 and Table S6 provide the complete mapping and harmonization contract.

### Gaussian interaction profile kernel similarity for mRNA and disease

Gaussian interaction-profile kernels represented similarity among mRNAs and among diseases [22]. For $A\in \{0,1\}^{p\times d}$, define the mRNA profile $I(m_{i})=A_{i:}$ and disease profile $I(d_{c})=A_{:c}$. The two similarities were


(1) \begin{align*} {\mathrm{SGM}}(m_{i},m_{j})&=\exp\{-\rho_{m}\lVert I(m_{i})-I(m_{j})\rVert^{2}\}, \end{align*}

(2) \begin{align*} {\mathrm{SGD}}(d_{c},d_{c^{\prime}})&=\exp\{-\rho_{d}\lVert I(d_{c})-I(d_{c^{\prime}})\rVert^{2}\}. \end{align*}

The normalized bandwidths were


(3) \begin{align*} \rho _{m}=\rho ^{\prime}_{m}/\{p^{-1}\sum _{i}\lVert I(m_{i})\rVert ^{2}\} \end{align*}

and


(4) \begin{align*} \rho _{d}=\rho ^{\prime}_{d}/\{d^{-1}\sum _{c}\lVert I(d_{c})\rVert ^{2}\},\end{align*}

with


(5) \begin{align*} \rho ^{\prime}_{m}=\rho ^{\prime}_{d}=1 \end{align*}

[23]. This gives $S_{M}\in \mathbb{R}^{p\times p}$ and $S_{D}\in \mathbb{R}^{d\times d}$. In every association fold, held-out positives were removed before both matrices were recomputed. Thus, neither a kernel nor the graph encoded the test edges.

Association ranking compared LAGCN with NMCMDA, SCPLPA, and DGP-PGTN under identical masked-edge folds and negative samples [24–26]. AUPR was the primary imbalance-sensitive metric, and AUROC was secondary. Precision at fixed retrieval budgets, alternative unknown-to-positive ratios, and positive–unlabelled analyses were descriptive sensitivities. Full masking, sampling, selection, and bandwidth-sensitivity rules are reported in Supplementary Method S1.

### Important mRNA identification through hybrid fusion

ImRIHF contains two connected modules (Fig. 1). LAGCN performs early fusion of the association matrix and the two Gaussian-kernel similarity matrices. It returns one cancer-specific score $a_{ic}\in (0,1)$ for each mRNA *i* and known parent cancer *c*. Intermediate fusion then combines that score with normalized patient expression. GCNDC constructs a patient graph from the resulting weighted expression and predicts subtype probabilities. LAGCN and GCNDC are evaluated at separate association-edge and patient-sample boundaries.

**Figure 1.**
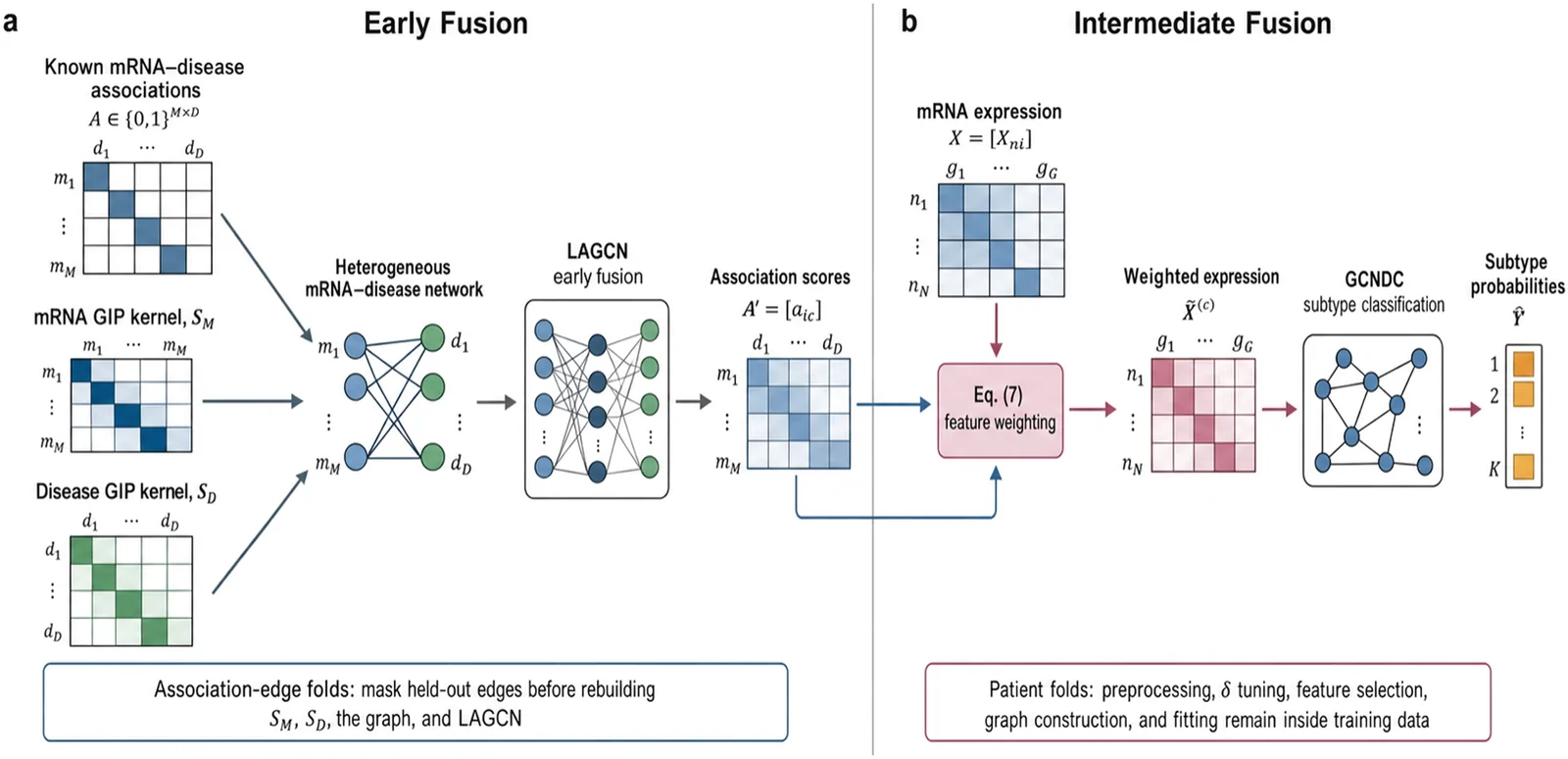
ImRIHF two-stage workflow and validation boundaries. Panel (a) shows the early-fusion chain from the association matrix and two Gaussian interaction-profile kernels through the heterogeneous graph and LAGCN to the association-score matrix. Panel (b) shows how expression and association scores enter Equation (7), weighted expression, GCNDC, and subtype probabilities. The lower bands distinguish association-edge and patient-sample evaluation boundaries.

#### Layer attention graph convolutional network

LAGCN propagates information over a normalized heterogeneous graph of mRNAs and diseases [27–30]. The graph has block form $G=[\begin{smallmatrix}S_{M}&A\\A^\top &S_{D}\end{smallmatrix}]$. Self-loops and symmetric degree normalization are applied before two graph-convolutional propagation layers. Node identity is the only input feature. Layer attention combines the input projection and both propagated representations. A bilinear decoder then produces the mRNA–disease logit $\eta _{ic}$.


(6) \begin{align*}& A^{\prime}=[a_{ic}]=\operatorname{LAGCN}(S_{M},A,S_{D}),\qquad a_{ic}=\operatorname{sigmoid}(\eta_{ic}).\end{align*}

The sigmoid score was used directly. No column-wise min–max scaling, rank normalization, or outcome calibration was applied. A zero decoder logit therefore gives $a_{ic}=0.5$, which is the neutral relevance score in Equation (7). Masked binary cross-entropy used only current edge-training positives and seed-fixed training unknowns. After edge-level selection, one score matrix was refitted from the frozen precutoff association object and locked before patient modelling. Patient expression and subtype labels never entered this refit. Supplementary Method S1 gives the layer equations, optimization settings, early stopping, and failure rules.

#### Intermediate fusion

Let $X_{ni}$ be normalized expression for mRNA *i* in patient *n*, and let $a_{ic}$ be its locked association score for parent cancer *c*. The cohort-wide index *c* is known before classification and is not the patient’s unknown subtype. The implemented transformation was


(7) \begin{align*}& \widetilde{X}_{ni}^{(c)}=2X_{ni}a_{ic}\exp\!\left(-\delta\lvert X_{ni}\rvert\right).\end{align*}

The multiplier $2a_{ic}$ makes 0.5 neutral on the locked score scale. The non-negative parameter *δ * attenuates extreme standardized expression. Equation (7) equals $X_{ni}$ at $a_{ic}=0.5$ only when *δ =0*. Because each patient in a cancer cohort receives the same score vector, the prior cannot directly encode subtype. It acts as a fixed diagonal rescaling, while subtype discrimination remains learned from training patients. Supplementary Method S1 and Table S2 give the transform rationale, alignment result, robustness families, and locked numerical traces.

GCNDC uses two graph-convolutional layers, ReLU after the first layer, and a softmax output trained by class-weighted cross-entropy. For each training partition, patient pairs were ordered by decreasing cosine similarity. The target mean degree was selected inside the inner loop, and the corresponding edge count fixed the training graph. The last retained similarity and a deterministic patient-hash tie-break defined the held-out attachment boundary. Training degrees and representations were frozen before any held-out patient was scored. A held-out patient could receive messages from itself and training anchors, but could not alter a training node or connect to another held-out patient. Supplementary Table S1G gives the complete algorithm.

For *n* training patients and target mean degree *γ *, the number of nonself edges was $E^\ast =\mathrm{min} \{ n({n-1})/2, \ \mathrm{round}(n\gamma /2)\}$. Self-loops were added only after membership was fixed. Binary-threshold graphs were primary. Cosine-weighted and mutual-*k*NN variants were evaluated at matched training mean degree. A sign reversal or absolute paired-effect drift above 0.005 restricted the conclusion to the primary binary construction.

Patient evaluation combined descriptive repeated refits with a separate locked audit. Twenty repetitions of stratified five-fold outer cross-validation, each containing five inner folds, described full-pipeline stability. Repetitions and folds were not treated as independent patients and supplied no probability to the computational master family. A class-stratified 70/30 development/audit split instead supplied one frozen prediction vector per audit patient. The audit totals were 100 for COADREAD, 129 for UCEC, 77 for SARC, 311 for BRCA, and 153 for LGG. UCEC allocated 90 EEA, 10 MSEAC, and 29 SEA patients to audit before fitting. Because only 10 MSEAC audit patients were available, UCEC remained feasibility-only.

Every data-dependent operation used development or current training patients only. This included expression preprocessing, feature ranking, graph construction, hyperparameter selection, stopping, and model fitting. The inner loop selected *δ *, feature count, target mean degree, and method-specific optimization settings. Configurations missing a required inner fold were ineligible. Exact grids, deterministic ordering, training budgets, and graph-construction sensitivities appear in Supplementary Method S1 and Tables S1B and S8.

The frozen *δ * grid was $0,10^{-4},3\times 10^{-4},10^{-3},3\times 10^{-3},10^{-2},3\times 10^{-2},10^{-1},0.3,1,$ and *3*. Target mean degrees were 2, 4, 8, 16, 32, and 64. GCN learning rates were $10^{-4},3\times 10^{-4},10^{-3},3\times 10^{-3}$, with widths 32, 64, and 128. Dropout was 0, 0.2, or 0.5, and weight decay was 0, $10^{-5}$, or $10^{-4}$. Training was capped at 300 epochs with patience 30.

Matched GCNDC was the primary mechanism comparator. It shared the association-eligible manifest, patient splits, preprocessing, graph rule, tuning budget, and classifier architecture with ImRIHF, but received neither association scores nor Equation (7). FullTx-EN and FullTx-GCNDC were the two prespecified full-transcriptome controls. Information-matched elastic net, Random Forest, Naive Bayes, GEDFN, Rhee-RN-GCN, ERGCN, GCNCC, and a clearly labelled GeSubNet-derived adapter provided additional descriptive context [17–21]. Association-ranking methods were not treated as patient-level subtype comparators.

READ-positive F1 was the primary metric for COADREAD, and unweighted macro-F1 was primary for UCEC, SARC, BRCA, and LGG. METABRIC and CGGA used macro-F1, while GSE14333 used rectum-positive F1. Accuracy, balanced accuracy, per-class recall, calibration, and prediction-set properties were secondary. The metric definitions, zero-denominator rules, comparator implementation contract, and coverage requirements are reported in Supplementary Method S1.

The computational family retained 29 permanently occupied coordinates. H01–H11 represent direct internal or strict-common external contrasts. S01–S07 represent internal full-transcriptome contrasts; K01–K05 represent disease-specificity contrasts; and X01–X06 represent external full-transcriptome contrasts. One Holm adjustment was applied across all 29 raw probabilities. A fixed-dimension one-sided max-*T* object supplied simultaneous lower bounds when the prespecified object was available. Coordinates could not be removed after results were observed. H02, H07, S02, and K02 remained occupied adverse UCEC feasibility slots with raw and adjusted *p=1* and no simultaneous lower bound.

For a computable coordinate, the independent unit was a patient unless the release manifest defined a larger dependence cluster. The effect was the paired difference in the fixed F1 or macro-F1 metric. Paired whole-cluster influence contributions generated studentized Rademacher compatibility probabilities. Shared patients used the same multiplier stream across comparisons. Supplementary Method S1 reports the full estimand, quality gates, Monte Carlo envelope, Holm calculation, max-*T* construction, and interpretation map. Supplementary Tables S4A–S4E give the corresponding coordinate records. No aggregate master-family passage statement was made.

The invariant statistical boundary was zero. A coordinate could support its mapped retrospective wording only when its conservative master-Holm value was below 0.05, its conservative simultaneous lower bound exceeded zero, and no mapped quality veto fired. The displayed 0.010, 0.015, and 0.020 margins were historically informed engineering sensitivities and did not define passage. Diagnostics could remove an interpretation but could not add one.

External transfer excluded the locked internal-audit partition. Within the remaining development data, a fixed identifier hash created a model-development core and a reserved uncertainty pool. The pool was divided into disjoint temperature-fitting and conformal-threshold subsets. Model selection and fitting used the core only, and the resulting predictor was never refitted after either calibration step. The same frozen predictor, temperature, feature map, graph rule, and thresholds were then applied to each external patient. Predictions were sealed before external labels were joined.

An external analysis was ineligible when >5% of its required selected genes were absent after the prespecified probe and gene mapping. Otherwise, absent selected features received the frozen development mean and became standardized zero. A second development-frozen empirical reference quantile provided a no-target-fit harmonization sensitivity. It could restrict, but could not rescue, the primary transfer interpretation.

The strict-common external analysis gave both graph methods one shared annotation-defined universe. FullTx-EN and FullTx-GCNDC retained their separately labelled native universes. H09–H11 therefore quantify strict-common matched-GCNDC transfer in METABRIC, CGGA, and GSE14333, whereas X01–X06 quantify the two full-transcriptome controls. External scopes were not pooled or substituted after scoring. Supplementary Tables S3A–S3C and S6 report mapping, calibration, coverage, and prediction-set audits.

Robustness analyses tested whether an apparent advantage depended on score identity, generic curation, transform choice, graph construction, prior source, graph topology, or target-processing artefacts. These analyses included matched score-map permutations, continuous matched-marginal weights, alternative transform families, degree-preserving rewiring, prior-edge perturbation, alternative-prior reconstruction, patient-label permutation, and a cross-platform negative control. They were prespecified vetoes outside the 29-coordinate family and could suppress, but never create or rescue, a performance interpretation. Their algorithms and result-specific limits are reported in Supplementary Method S1 and Tables S1D–S1F and S5.

Association-object construction, patient-model fitting, prediction generation, label joining, and scoring were separated operationally. Audit and external outcomes were not used during model fitting or prediction generation, and no stage was repeated after downstream outcome access. This boundary limits execution-time adaptation but cannot erase historical knowledge of public cohorts or earlier analyses. All reported comparisons therefore remain retrospective and source-conditional. They do not establish prospective confirmation, population generalization, clinical utility, or a biological mechanism.

## Results

### Association-score prediction

LAGCN produced a cancer-conditioned association-score matrix with mRNAs in rows and diseases in columns. Figure 2a displays fixed subsets of the COADREAD, UCEC, and SARC columns. The downstream analysis used the bilinear decoder’s sigmoid score $a_{ic}\in (0,1)$ directly, without columnwise rescaling or post-hoc calibration. Association-edge folds were distinct from the patient-sample folds used for classification. In every edge fold, held-out positive associations were removed before the Gaussian interaction-profile kernels and heterogeneous graph were rebuilt.

**Figure 2.**
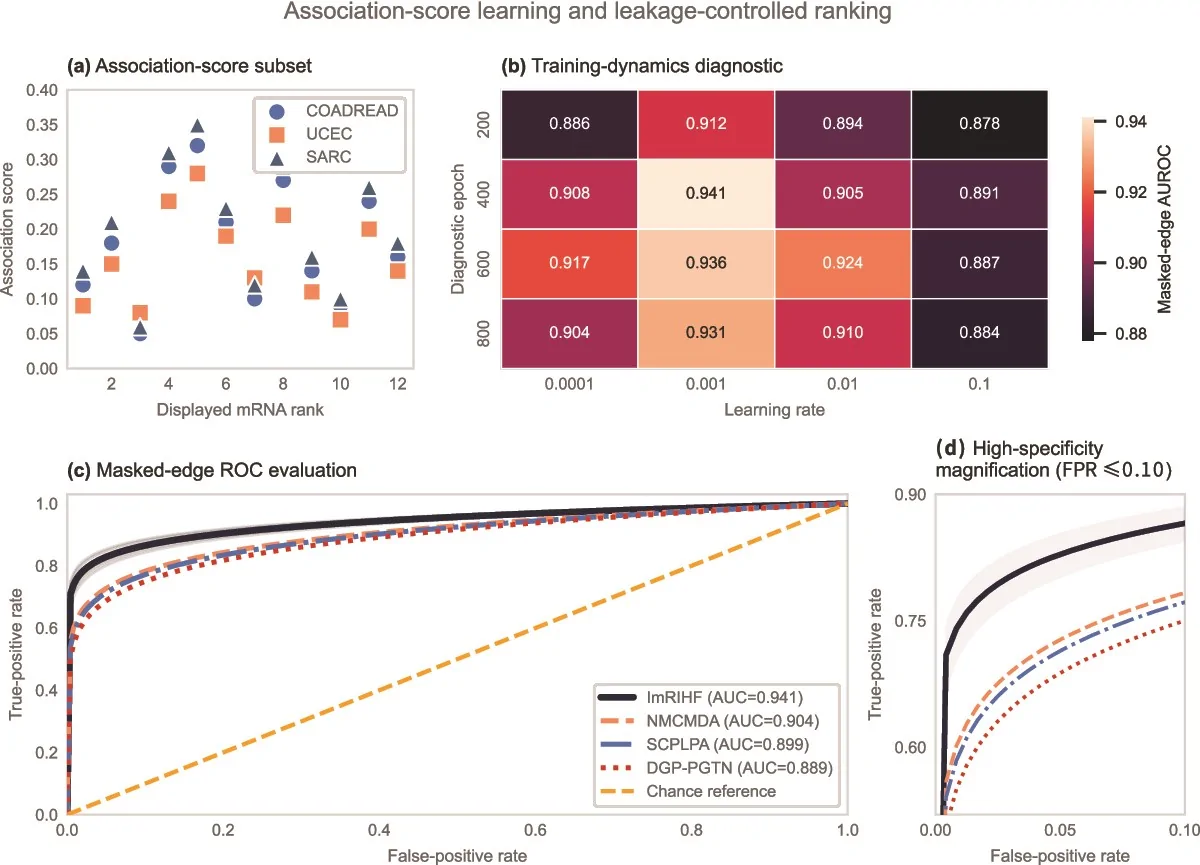
Association scoring and leakage-controlled evaluation. (a) Fixed-size subset of disease-specific association scores; the displayed ranks are not the complete feature universe. (b) Descriptive training-dynamics diagnostic that did not determine the AUPR-based final configuration. (c) ROC curves from folds in which held-out edges are masked before both Gaussian kernels and the heterogeneous graph are rebuilt. (d) A separate magnification of the prespecified high-specificity region of panel (c) (false-positive rate *≤ 0.10*), shown outside the main ROC axes to avoid obscuring data. All methods use identical folds and negatives.

The archived association-ranking estimate was AUC 0.9595, but it was not treated as leakage-controlled evidence. After edge masking, the concatenated held-out predictions yielded AUROC 0.941. Fold-specific AUROCs were 0.931, 0.935, 0.941, 0.948, and 0.950, giving a mean of *0.941± 0.008*, where *± * denotes the sample standard deviation. Under identical folds and negative samples, AUROC was 0.904 for NMCMDA, 0.899 for SCPLPA, and 0.889 for DGP-PGTN [24–26]. AUPR was the primary imbalance-sensitive metric. In the primary 1:1 degree-matched analysis, ImRIHF and NMCMDA achieved AUPR values of 0.932 and 0.887, respectively. At a retrieval budget of 50 pairs, the corresponding precision values were 38/50 (0.76) and 32/50 (0.64). ImRIHF AUPR remained 0.781 and 0.674 at unknown-to-positive ratios of 5:1 and 10:1.

Dependence-aware folds preserved the same ordering. Evidence-source-disjoint analysis yielded AUPR 0.902 versus 0.864, a difference of 0.038 with cluster interval 0.022–0.054, and AUROC 0.918 versus 0.889, a difference of 0.029 with interval 0.017–0.041. Publication-disjoint AUPR and AUROC differences were 0.039 and 0.030, while entity-disjoint differences were 0.036 and 0.028. Complete denominators, group counts, and intervals are reported in Supplementary Table S1A (continued). These grouped analyses reduce dependence from shared sources, publications, or entities, but they do not make the curated prior independent of the literature.

The temporally separated analysis contained 90 newly supported positives among 414 eligible pairs. The high-score group contained 50 supported and 100 unsupported pairs, compared with 40 supported and 224 unsupported pairs in the reference group. The resulting odds ratio was 2.80, with a Wald 95% interval of 1.74–4.52 and exploratory-screen BH *q=0.012*. This analysis tested later association support and did not constitute independent patient-level validation. Prior-perturbation, alternative-prior, and disease-specificity results are reported in Supplementary Tables S1D, S1E, and S4D and Figs S1 and S2.

### Internal cancer-classification results

Feature weighting was next applied to release-level mRNA expression for cancer classification. The primary metric was READ-positive F1 for COADREAD and unweighted macro-F1 for UCEC, SARC, BRCA, and LGG. Accuracy, weighted F1, AUROC, balanced accuracy, class recall, and calibration were secondary where applicable. Figure 3 summarizes 20 repeated full-pipeline refits for the prespecified internal metrics and selected comparators. Each repeat contained complete nested preprocessing, feature selection, tuning, graph construction, and outer prediction. The distributions describe stability across refits; the repeats are not independent patients and do not provide H01–H08 estimates or probabilities.

**Figure 3.**
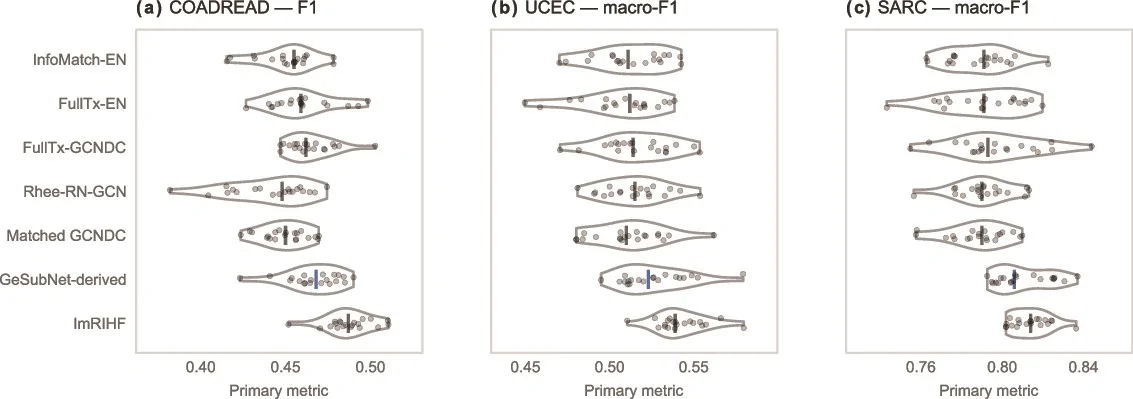
Repeated full-pipeline internal-cohort stability. The three primary-metric panels compare ImRIHF with matched GCNDC, FullTx-GCNDC, FullTx-EN, InfoMatch-EN (information-matched elastic net on the association-eligible manifest), Rhee-RN-GCN, and the GeSubNet-derived-score adapter. ACC, AUROC, weighted F1, Random Forest, Naive Bayes, GEDFN, ERGCN, and GCNCC remain in the broader supplementary comparator matrix. Each distribution comprises 20 full-pipeline repeat summaries and is descriptive; repeats are not independent patients. H01–H08 use separate locked-audit prediction pairs, not these distributions. The GeSubNet-derived-score adapter remains a modified descriptive endpoint, and its SARC interval crosses zero.

The locked audit answered a separate question. One frozen prediction vector from each method was evaluated for every audit patient, using audit totals of 100, 129, 77, 311, and 153 for COADREAD, UCEC, SARC, BRCA, and LGG, respectively. The H01–H05 ImRIHF-minus-matched-GCNDC effects were *+0.034*, *+0.032*, *+0.027*, *+0.032*, and *+0.035*. The corresponding ImRIHF/control primary metrics were 0.473/0.439, 0.551/0.519, 0.798/0.771, 0.729/0.697, and 0.744/0.709 in cohort order. For COADREAD, the marginal interval was 0.025–0.049 and raw *P=.0005*. For SARC, the interval was 0.019–0.029 and raw *P=.0005*. BRCA and LGG intervals were 0.018–0.040 and 0.023–0.041, with raw *P=.0030* and 0.0010, respectively. These effects came from the frozen audit predictions and not from the central values of the repeated-CV distributions.

UCEC was restricted before fitting to an audit allocation of 90 EEA, 10 MSEAC, and 29 SEA patients, with 221, 11, and 69 in development. Its matched-GCNDC effect was *+0.032*, but the 10 MSEAC audit units did not meet the prespecified inferential qualification. H02, H07, S02, and K02 therefore retained raw and master-adjusted *p=1* and no joint lower bound, irrespective of their observed effects. UCEC remained a feasibility analysis and did not support a cohort-level or multi-cohort inferential statement.

The repeated full-pipeline medians for ImRIHF were 0.487, 0.539, 0.814, 0.721, and 0.759 in cohort order. The corresponding ImRIHF-minus-matched-GCNDC median differences were 0.037, 0.029, 0.024, 0.029, and 0.032. These values were close in scale to, but numerically distinct from, the locked effects. The broader repeated-CV comparator matrix retained FullTx-GCNDC, FullTx-EN, InfoMatch-EN, Rhee-RN-GCN, the GeSubNet-derived-score adapter, and additional conventional and graph baselines. Complete secondary metrics are reported in Supplementary Table S4C.

Full-transcriptome controls tested whether a favourable comparison arose from restricting the gene universe. Locked ImRIHF-minus-FullTx-EN differences were 0.028, 0.027, 0.023, 0.020, and 0.019 across COADREAD, UCEC, SARC, BRCA, and LGG. The corresponding FullTx-GCNDC differences were 0.025, 0.025, 0.021, 0.017, and 0.015. UCEC FullTx coordinates remained descriptive under the same rare-class restriction. Supplementary Tables S4A–S4B report the audit values, intervals, raw probabilities, and coordinate roles. No comparator was selected from audit or outer-test performance.

Class-recall changes were distributed across endpoint classes rather than confined to one majority class. The complete class-specific array is reported in Supplementary Table S1F. The MSEAC value remained descriptive because the locked UCEC audit contained 10 cases.

The five tasks also had different evidential roles. COADREAD was an anatomical-site benchmark, while SARC was a histopathological-subtype task. BRCA reconstructed expression-derived PAM50 labels after excluding label-defining genes and therefore did not provide independent molecular validation. LGG used the DNA-defined IDH/1p19q endpoint and was the stronger label-independent molecular task. These distinctions limited every result to its named endpoint.

### External transfer

The deployment-core BRCA and LGG predictors were transferred to METABRIC and CGGA without outcome-informed refitting, target-cohort normalization, feature substitution, threshold adjustment, or external-label tuning. The locked 30% internal-audit partitions did not enter the transfer objects. Each external patient connected only to frozen development anchors. The primary external contrast used a strict-common annotation-defined graph universe shared by ImRIHF and matched GCNDC, whereas FullTx controls retained separately labelled native and portability universes.

In METABRIC, strict-common macro-F1 was 0.673 for ImRIHF and 0.639 for matched GCNDC. H09 was therefore *+0.034*, with a marginal interval of 0.023–0.045 and raw *P=.0028*. In CGGA, the corresponding values were 0.707 and 0.681. H10 was *+0.026*, with interval 0.019–0.033 and raw *P=.0075*. Native ImRIHF mapping was 249/256 genes in METABRIC and 247/256 in CGGA. External calibration, prediction-set measurements, and method-specific mapping denominators are reported in Supplementary Tables S3A–S3B and Fig. S4.

GSE14333 evaluated the endpoint-matched colon-versus-rectum transfer. After excluding one record without a location label, 250 colon and 39 rectal tumours remained. On the 246-gene strict-common graph universe, rectum-positive F1 was 0.703 for ImRIHF and 0.667 for matched GCNDC. H11 was *+0.036*, with interval 0.023–0.049 and raw *P=.0035*. The observed TP/FP/FN/TN cells were 26/9/13/241 and 23/7/16/243, respectively. FullTx-EN and FullTx-GCNDC remained mandatory controls on the same patients but did not enter the strict-common H contrast.

The native-universe ImRIHF-minus-FullTx-EN effects were 0.029, 0.025, and 0.033 in METABRIC, CGGA, and GSE14333. The corresponding FullTx-GCNDC effects were 0.027, 0.023, and 0.030. Supplementary Table S3C identifies these as X01–X06. The coordinate-level effects are reported for the three named cohorts without an aggregate competitive-transfer or master-family passage claim. Each endpoint had one retrospective external cohort, so these results did not establish endpoint-wide reproducibility, population generalization, or clinical utility.

Calibration and uncertainty summaries used the unchanged deployed ImRIHF predictor. NLL, Brier, ECE$_{15}$, and empirical prediction-set measurements are reported in Supplementary Table S3B and Fig. S4. These source-conditional measurements did not provide a finite-sample coverage guarantee after cross-cohort distribution shift.

GSE21050 remained an endpoint-mapped descriptive sensitivity because its pooled-undifferentiated category was only partially lineage matched. Strict-common macro-F1 was 0.742 versus 0.716, a difference of *+0.026* with bootstrap interval 0.017–0.035 and nominal *P=.0090*. GSE17025 lacked MSEAC and could not validate three-class UCEC; its separately labelled EEA-versus-SEA result was descriptive. Full source-specific, label-coarsening, mapping, and calibration results are retained in Supplementary Tables S3B and S3D.

### Ablation and robustness

The factorized ablation crossed real versus covariate-matched score maps with damping absent (*δ =0*) versus damping present, selected independently within each inner loop. It also retained the unweighted reference. The full model remained above the unweighted and median matched-permutation references in COADREAD, UCEC, and SARC. Figure 4 displays the complete five-cell sequences.

**Figure 4.**
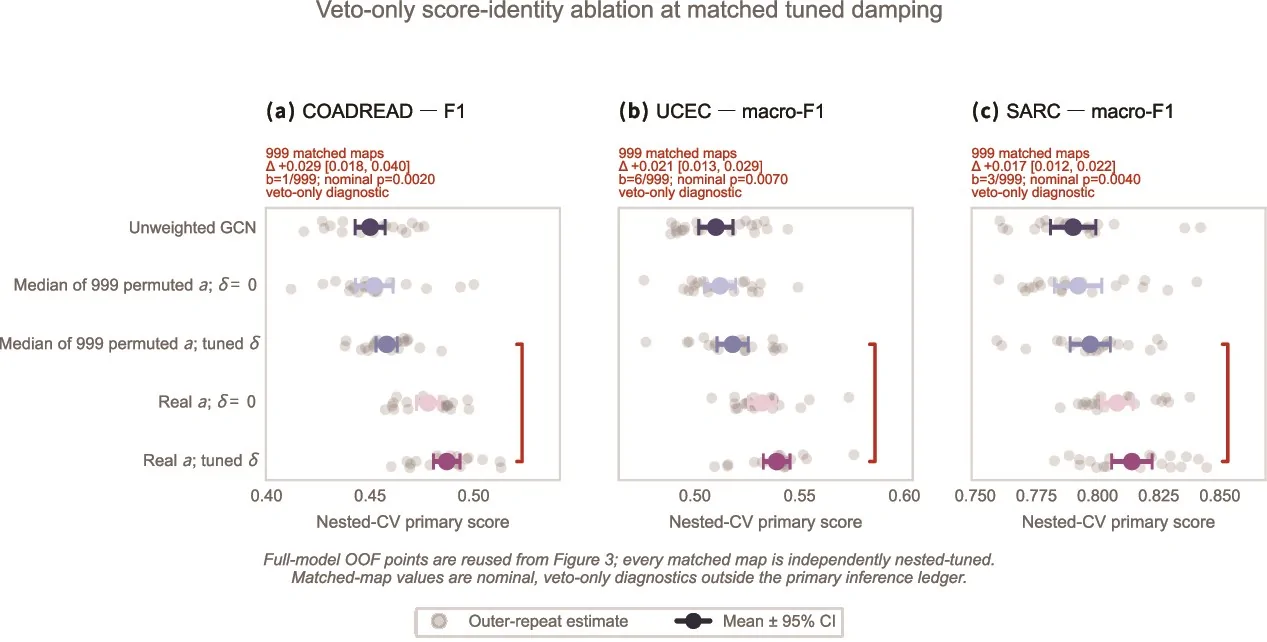
Factorized score-by-damping ablation. Each cohort includes an unweighted reference and real-score cells at *δ =0* and independently tuned *δ *; permuted cells are medians across 999 complete covariate-matched score-map pipelines using the same frozen grids. Brackets identify the prespecified real-map versus median independently tuned matched-permutation diagnostic. The full real-map out-of-fold predictions are reused, and dependent contrasts are not independent replications. Nominal diagnostic exceedance probabilities are shown without master identifiers or master-Holm labels because gene-score exchangeability is not a design fact.

The prespecified score-identity contrast compared the real map with the median of 999 complete, independently tuned, covariate-matched score-permutation pipelines. Differences were *+0.029* for COADREAD, *+0.021* for UCEC, and *+0.017* for SARC. Their split-sensitivity intervals were 0.018–0.040, 0.013–0.029, and 0.012–0.022, with nominal diagnostic *P=.002*, 0.007, and 0.004. These diagnostics were veto-only, did not enter the 29-slot family, and could not create or rescue a patient-level efficacy statement.

Additional robustness analyses tested prior deletion and addition, alternative prior sources, transformation choice, matched-marginal weights, graph construction, doubled search budgets, and small-class handling. At 20% prior perturbation, every displayed performance decrease was no greater than 0.030, topology advantage remained positive, and top-20 Jaccard was at least 0.40. Exponential, rational, and saturating transforms preserved positive effects, as did binary, cosine-weighted, and mutual-*k*NN graphs. Complete values and the limits of these retrospective diagnostics are reported in Supplementary Method S1, Tables S1B and S1D–S1F, S4D, and S5, and Fig. S2.

Source reconstruction from DisGeNET and Open Targets produced effects of *+0.031/+0.027/+0.024/+0.028/+0.030* across COADREAD/UCEC/SARC/BRCA/LGG. Feature-rank Spearman correlations with the RNADisease analyses were 0.47/0.45/0.43/0.44/0.49, and top-20 Jaccard values were 0.481/0.429/0.429/0.481/0.538. The direct cancer-prior-minus-strongest-control K gaps were 0.023/0.020/0.017/0.021/0.024. K02 remained the structural UCEC adverse slot with raw and master-adjusted *p=1* and no lower bound. These results tested source and curation specificity but could not rescue a failed performance coordinate.

Doubling the search budget left the method ranking unchanged, and every paired-gap drift rounded to 0.000. The complete cohort array is reported in Supplementary Table S1B. This descriptive analysis did not define a post-outcome strongest comparator.

The damping coefficient was selected only inside each inner fold. Cohort-level response curves peaked between 0.01 and 0.03, but the displayed peak was not imposed on every outer split. The complete *δ * response curves are presented in Supplementary Fig. S7 because they describe tuning behaviour rather than a separate outcome. Representative locked prediction traces remain in Supplementary Table S2 and Fig. S5. They illustrate complete-vector probability changes without attributing any change to one displayed gene.

### Historical candidate-gene audit

The candidate analysis was kept separate from the predictive comparison. The fixed historical challenge subset comprised ITGB4, DPP7, PGAM1, TRAPPC6A, RPL35A, IBSP, RHOC, DEK, LY6E, RALA, BAG3, and MCM6. It was drawn from earlier outcome-exposed candidate lists and was not the complete submitted candidate universe. Every mapped, unmapped, concordant, discordant, significant, and nonsignificant row remained in the denominator. The archived recurrence counts and the literature-context table are presented in Supplementary Table S7C because they provide historical context rather than independent validation.

In the external-expression audit, 11 of the 12 genes mapped and MCM6 remained platform-unmapped. Five mapped genes were direction-concordant, and four met BH *q<0.05*: ITGB4, PGAM1, DEK, and BAG3. Only DEK and BAG3 were both direction-concordant and BH-significant. Patient-level distributions, directed Hedges *g* estimates, mapping status, and the complete 12-row accounting are reported in Supplementary Fig. S6 and Tables S7A–S7B. Because the same public cohorts also assessed prediction, these findings provide secondary transcriptomic concordance rather than independent biological validation, an unbiased panel yield, or a mechanistic claim.

The fixed directed contrasts and 12-row BH procedure are detailed in Supplementary Method S1. MCM6 remained in the family with *p=q=1*, and predictive and expression endpoints from the same public cohorts were not combined.

## Discussion

The locked retrospective audit shows positive displayed F1 or macro-F1 point differences between the frozen prior-weighted prediction rule and its component and full-transcriptome comparators in the named cohorts, conditional on the training data, fitted models, class set, and prediction rule. The study reports verified coordinate-level quantities without an aggregate master-family passage claim; the four fixed UCEC structural slots remain feasibility-only and gate-ineligible by design. The results do not establish generic method superiority, retraining uncertainty, prospective confirmation, or study-programme-wide type-I error control. The nonzero historical margins were designed after outcome access and are reported only as sensitivity values. UCEC is a three-class feasibility cohort and cannot support cohort-level or multi-cohort inferential wording. BRCA is a secondary expression-derived PAM50 reconstruction benchmark even after the defining genes are excluded; LGG is the stronger DNA-defined, label-independent molecular-subtype task. SARC remains a histopathologic task, and COADREAD remains a separately named anatomical-site stress test. Earlier work established network-constrained expression models, patient-similarity GCNs, multi-omics integration, subtype-specific network inference, and general reproducibility reporting. The contribution here is the auditable cross-task transfer estimand and its biology-specific separation of association-edge, patient-sample, candidate-selection, and external-cohort boundaries. The alignment proposition applies at any fixed damping value and explains when diagonal prior weighting can improve a separability surrogate; it does not replace held-out predictive evidence. The deliberately modified GeSubNet-derived-score adapter is descriptive, does not support a SARC advantage, and supports no statement about native GeSubNet or subtype-network-generation methods as a class. The evidence also defines strict boundaries. METABRIC, CGGA, GSE14333, and GSE21050 were patient-disjoint computational transfers, but each is reported only as a named-cohort transfer result; no composite transportability passage is asserted. One historically accessible external cohort per endpoint cannot establish endpoint-level reproducibility, multi-site robustness, or population generalization. GSE17025 lacks MSEAC and cannot validate three-class UCEC. The SARC pooled-other mapping is only partially lineage matched. Candidate-expression and prediction analyses that use the same public cohort are non-independent endpoints from one sample and are never combined. The fixed historical 12-gene challenge subset and its public external-expression results remain subject to winner-selection bias and do not establish a general biological mechanism, validate Equation (7), demonstrate clinical utility, or prove causality. RNADisease and other curated resources remain incomplete and potentially correlated; the master K coordinates and the attention-matched, technical-measurability, wrong-disease, and alternative-prior controls bound rather than eliminate this limitation. ‘Interpretable’ denotes a transparent cancer-conditioned metric, audited feature weights, and reconstructable prediction traces; it does not mean that every selected gene is biologically stable or causal. Claim-specific failure rules prevent one result from rescuing another. A failed or ineligible master analysis remains in its fixed slot with raw and master-adjusted *p=1*, and a failed association, full-transcriptome comparator, score-alignment, graph, source-reconstruction, curation, or technical gate removes every dependent interpretation in the interpretation map. A failed transfer or X coordinate removes only that named competitive-transfer statement; public candidate-expression evidence cannot create or rescue a computational comparison. Numerical nonconvergence is not repaired by seed or configuration substitution. The contribution is a descriptive locked retrospective single-transcriptome analysis of disease-association-guided weighting plus a separately bounded audit of a historical 12-gene challenge subset. No *de novo* discovery, prospective confirmation, clinical-utility, or health-economic claim is made.

## Conclusion

Under leakage-contained association-edge, patient-sample, and external-transfer boundaries, the frozen disease–gene prior yielded modest positive retrospective differences from matched controls in the named cohorts. The separate audit of a historical 12-gene challenge subset provided limited transcriptomic concordance rather than de novo biomarker validation. These findings motivate prospective and independently replicated evaluation of disease-association-guided single-transcriptome classification; they do not establish generic superiority, clinical utility, causality, or population generalization.

**Key Points**

- Early fusion uses a layer attention graph convolutional network to learn mRNA-disease priors for feature weighting.
- Intermediate fusion uses a graph convolutional network for disease classification with feature weights and learned priors for subtype classification.
- The study reports conditional paired comparisons between the frozen ImRIHF and comparator prediction rules in named cohorts and audits a historical 12-gene mRNA challenge subset; the retrospective results do not establish de novo biomarker validation.

## Supplementary Material

Supplementary_material_elag009

## Data Availability

Data and code are available at https://github.com/Jie-Ni/ImRIHF.
