# Supplementary material for: Knowledge-guided graph fusion of mRNA profiles for interpretable cancer subtyping
Source: Brief Funct Genomics. 2026 Oct 1;25:elag009. doi: 10.1093/bfgp/elag009 (PMC13630462; doi:10.1093/bfgp/elag009)
Supplement: Supplementary file 1 [file supplementary_material_elag009.zip › BFGP-26-0044_Supplementary_Information.pdf]

Supplementary Information

Knowledge-Guided Graph Fusion of mRNA Profiles for Interpretable Cancer Subtyping

Supplementary Method S1. Detailed methods and statistical analysis

Association-edge containment

For every association-edge outer fold, the held-out positive edges are removed before Gaussian interaction-profile kernels are computed. The source-first association universe starts from the complete pre-cutoff RNADisease record after the declared evidence and cohort-source exclusions, maps every mRNA identifier independently of archived expression-panel membership, and retains all mapped RNADisease mRNA nodes. The archived 9,182-edge/4,928-mRNA object serves only as provenance and does not define the final universe. At each patient fitting boundary, the usable association vector is the intersection of the source-derived nodes with the current training-derived technical-pass anchor universe $\mathcal{U}$; no held-out expression value, held-out label, archived 2,000-mRNA panel, archived 4,928-mRNA membership, or global expression-occurrence list defines it. The final edge denominator is derived from the source-first object rather than being forced to 9,182. Each held-out positive denominator is paired with an equal number of seed-fixed, role-deduplicated, degree-matched unknown edges. Within the outer-training graph, five edge-stratified inner folds repeat masking before both kernels. Hyperparameters maximize mean inner AUPR; ties within 10^{-12} use mean inner AUROC and then the lower identifier in a hashed canonical configuration list. After the score model is fixed, the decision threshold maximizes mean inner F1 over 0.3/0.4/0.5/0.6/0.7, with the lower threshold winning a tie; it cannot affect AUROC, AUPR, or precision@50. The forbidden-input audit confirmed that no held-out edge contributed to a kernel, graph, hyperparameter, checkpoint, or score. The association AUROC and AUPR use only concatenated outer-held-out predictions from the complete source-first denominator. This boundary is independent of the patient-sample boundary.

Gaussian-kernel bandwidth sensitivity. The primary scale constants remain $\rho'_d = \rho'_m = 1$, matching the archived implementation. Frozen sensitivity runs repeat the complete association and downstream pipelines at joint values 0.5 and 2.0. Patient outcomes cannot select a bandwidth. A cohort-level knowledge-guidance interpretation is retained only if both sensitivities preserve its effect direction and change the paired difference by no more than 0.005.

Nested patient-sample evaluation

The descriptive stability schedule comprises 20 repeated stratified fivefold outer splits applied to release-level unselected expression. Archived 2,000-mRNA panels and ranks are excluded from every new predictive, inferential, and candidate-ranking analysis and appear only as explicitly labelled historical context. The patient-level list is InfoMatch-EN, Random Forest, Naive Bayes, FullTx-EN, FullTx-GCNDC, GEDFN-multiclass, Rhee-RN-GCN, ERGCN-one-way-inductive, GCNCC-multiclass, GCNDC without association weights, GeSubNet-derived-score, and ImRIHF. InfoMatch-EN and the other information-matched methods use the source-first, training-boundary association-eligible manifest. FullTx-EN and FullTx-GCNDC instead begin from every technically eligible release-level gene and receive no association matrix, graph-derived filter, Equation (7), or archived panel. Native-universe and platform-portability rows remain visible for every method even when unfavourable or ineligible. The strict-common graph row applies only to ImRIHF and matched GCNDC on one shared anchor universe; FullTx controls retain separately labelled native and portability scopes. Repeated folds provide no master-family probability or patient-sampling confidence bound.

The cohort estimand preserves the locked patient manifests while using official release-level unselected expression in place of the archived 2,000-mRNA feature panels. Mapping assigns one released primary-tumour expression record one-to-one to every locked participant: 332 COADREAD patients (254 COAD/78 READ), 430 UCEC patients (311 EEA/21 MSEAC/98 SEA), and 257 SARC patients (58 DDLPS/103 LMS/96 in a frozen enumerated pooled-other histology list). It yielded zero unresolved identifiers, duplicate participants, non-primary records, missing endpoint labels, endpoint-incompatible records, or post-mapping release-level QC failures. These are observed release-level denominators. Eligibility is adjudicated separately for each cohort. A nonzero unresolved discrepancy retains every affected master slot with effect and interval NA and raw and master-adjusted $p = 1$, stops the candidate stream and downstream transfer object, and permits no archived substitute or replacement patient. BRCA uses the four core PAM50 classes and excludes Normal-like cases and all PAM50 predictors. Because PAM50 is expression-derived, this exclusion prevents direct defining-panel reuse but does not create a transcriptome-independent or clinical endpoint; BRCA is an expression-label benchmark. LGG uses the frozen DNA-defined IDH/1p19q endpoint and excludes label-defining genes from candidate evidence. The final BRCA and LGG denominators are 1,036 and 511 patients, respectively.

For H01–H08, each internal cohort receives one identifier-hash, class-stratified 70/30 development/audit split. UCEC is additionally constrained before fitting: after exact reconciliation of the final 311 EEA/21 MSEAC/98 SEA singleton units, ten MSEAC audit places are fixed first and Hamilton largest-remainder allocation of the other 119 places gives 90 EEA and 29 SEA; development is 221/11/69. Within each class, identities are ordered by the public SHA-256 hash domain separator `BFGP-26-0044|UCEC|locked-audit-v1`. The canonical UTF-8/NFC input fields are the domain separator, class label, and normalized public independent-unit identifier, joined by one `|`; rows are sorted by the digest as an unsigned big-endian integer and then by normalized-identifier UTF-8 byte order. UCEC confusion-component means and influence contributions use class proportions from the complete eligible release manifest and per-audit-unit weights $\pi_k / n_{k,\text{audit}}$, so the deliberate MSEAC audit oversampling does not redefine the class standardization. The ten-MSEAC allocation is a transparent descriptive audit: H02, H07, S02, and K02 have raw and master-adjusted $p = 1$ and no simultaneous lower bound by design, irrespective of the realized effect or simulation result. The current UCEC release cannot support inferential qualification because its locked audit contains only ten independent MSEAC units; refill, reassignment, replacement, and post-output extension cannot alter this limitation. Every data-dependent operation uses the development partition only. Each locked audit patient contributes exactly one frozen probability vector per compared method. H01–H05 compare ImRIHF with matched unweighted GCNDC in COADREAD, UCEC, SARC, BRCA, and LGG; H06–H08 compare ImRIHF with prespecified FullTx-GCNDC in COADREAD, UCEC, and SARC. The labels and earlier results were historically available, so the split is a retrospective audit rather than an outcome-naive test. Site, plate, batch, specimen source, and preservation fields are frozen where available; group-held-out and cluster-bootstrap sensitivities remain visible.

Within each training boundary, the declared scale is applied as $\log_2(\text{CPM}+1)$ for raw counts, $\log_2(x+1)$ for nonnegative abundance, or unchanged documented $\log_2$ data. Genes fail at greater than 5% missingness, raw-count CPM at least 1 in fewer than 10% of training patients, or zero post-transform training IQR. Those rules define the training-only technical-pass anchor universe $\mathcal{U}$ before association intersection or feature ranking. Each sample receives within-sample midranks and normal scores over all observed genes in $\mathcal{U}$; the rank transform is never recomputed on a selected subset. Frozen training-gene means and scales are then applied, and only afterward are the source-first association intersection, ANOVA-F rank, and requested feature list extracted. Held-out missing selected entries receive the corresponding frozen training mean before standardization. ANOVA-F ranking uses a lexicographic version-stripped Ensembl tie-break and $k \in \{64, 128, 256, 512\}$. The inner objective is READ-positive F1 for COADREAD and unweighted macro-F1 otherwise. Configuration ties within 10^{-12} use a frozen integer order; incomplete inner candidates fail. Epoch-trained outer fits use the ceiling of the median best inner epoch.

Operationally, the anchor-universe rank transform is completed before selected features are extracted,

and selected-subset reranking is forbidden.

Performance-metric definitions

For N evaluable predictions, accuracy is $\mathrm{ACC} = N^{-1} \sum_{i=1}^{N} I(\widehat{Y}_i = Y_i)$. For class k, let TP_k, FP_k, and FN_k be one-versus-rest counts and define $\mathrm{F1}_k = 2\mathrm{TP}_k/(2\mathrm{TP}_k + \mathrm{FP}_k + \mathrm{FN}_k)$. Binary F1 is $\mathrm{F1}_+$ for the positive class fixed before fitting: a known association edge for the association task, READ for every COADREAD contrast (H01, H06, S01, and K01), and rectum for every GSE14333 contrast (H11, X05, and X06). H02–H05 and H07–H10 use unweighted macro-F1. This metric map is immutable across the estimand, simulation, tables, thresholds, and claims. If $N = 0$ or an applicable F1 denominator is zero, the metric is undefined (NA); it is never silently set to zero. With $N_k = \sum_i I(Y_i = k)$ and a frozen K-class set, weighted F1 is $\sum_{k=1}^{K} (N_k/N)\mathrm{F1}_k$, whereas macro-F1 is $K^{-1} \sum_{k=1}^{K} \mathrm{F1}_k$; both are NA if any required class-specific F1 is undefined. Binary AUROC is $\Pr(s_+ > s_-) + \frac{1}{2}\Pr(s_+ = s_-)$ for the continuous positive-class score, estimated over all positive–negative pairs. Multiclass AUROC is the unweighted mean of the K one-versus-rest AUROCs. AUROC is NA if a required comparison has no positive or no negative observation. These conventions apply to every reported value.

Comparator adjudication determined that GeSubNet's native interaction endpoint is not a patient-classification benchmark and therefore is not used for a patient-level performance claim. The native-endpoint review and source-disjoint reference search also establish that the derived-score adapter is not a substitute for a native benchmark. Accordingly, no superiority or state-of-the-art claim over subtype-network-generation methods as a class is made. Within outer training, the deliberately modified derived-score adapter computes $s_i^{\mathrm{GS}} = \{K(|\mathscr{C}| - 1)\}^{-1} \sum_k \sum_{j \in \mathscr{C}, j \neq i} \hat{e}_{ij}^{(k)}$ and $a_i^{\mathrm{GS}} = \{\mathrm{rank}_{\mathscr{C}}(s_i^{\mathrm{GS}}) - 0.5\}/|\mathscr{C}|$; an uncovered eligible gene receives neutral $a_i = 0.5$. No held-out patient or label enters this score. The adapter is exploratory and cannot support a claim about native GeSubNet. Methods share folds, deterministic seeds, training-only boundaries, trial ceilings, and hardware-hour ceilings but retain native axes. Published defaults are trial 0. The outer test split is scored once and cannot affect preprocessing, tuning, stopping, graph construction, or panel selection.

For p mRNAs and d diseases, the implemented LAGCN uses $G = \begin{bmatrix} S_M & A \\ A^\top & S_D \end{bmatrix}$, $\widetilde{G} = G + I_{p+d}$, and $\widehat{G} = \widetilde{D}^{-1/2}\widetilde{G}\widetilde{D}^{-1/2}$. With $H^{(0)} = I_{p+d}$, $Z^{(0)} = H^{(0)}W_{\mathrm{in}}$ and $Z^{(\ell+1)} = \mathrm{ReLU}\{\widehat{G}Z^{(\ell)}W_\ell\}$ for $\ell = 0, 1$. Node-wise softmax attention $\alpha_{v\ell} \propto \exp\{q^\top \tanh(W_a z_v^{(\ell)})\}$ combines $Z^{(0)}, Z^{(1)}, Z^{(2)}$. The decoder logit is $\eta_{ic} = z_i^\top R z_c$ and the cancer-conditioned score used downstream is exactly $a_{ic} = \mathrm{sigmoid}(\eta_{ic}) = (1 + e^{-\eta_{ic}})^{-1}$; no column-wise min–max normalization or outcome calibration is applied, and a zero logit gives the neutral score 0.5. Masked binary cross-entropy uses edge-training positives and an equal number of seed-fixed training unknowns. The inner-edge grid is width 64/128; dropout 0/0.2/0.5; Adam learning rate $10^{-4}/3\times10^{-4}/10^{-3}/3\times10^{-3}$; weight decay $0/10^{-5}/10^{-4}$; maximum epochs 200/300; patience 20/30; and decision threshold 0.3/0.4/0.5/0.6/0.7. Held-out positives are removed before both GIP kernels, graph construction, negative sampling, fitting, and selection.

LAGCN optimization and stopping. For LAGCN, the selected dropout is applied once to the first ReLU output before the second propagation layer. There is no input, skip-attention, or decoder-output dropout. Adam uses the selected learning rate and weight decay with $\beta_1 = 0.9$, $\beta_2 = 0.999$, and $\epsilon = 10^{-8}$. After every epoch, the frozen inner validation set is evaluated by mean AUPR. An improvement must exceed 10^{-6}, and patience counts consecutive nonimproving epochs. The earliest epoch attaining the best score is restored. An absent validation class, non-finite loss, or incomplete fold fails that configuration without a fallback. These optimizer and placement rules are part of the canonical configuration string and cannot be filled after held-out scores are observed.

The block dimensions fix the orientation unambiguously: $A \in \{0, 1\}^{p \times d}$ has mRNAs in rows and diseases in columns, $I(m_i) = A_{i:}$, $I(d_c) = A_{:c}$, $S_M \in \mathbb{R}^{p \times p}$, and $S_D \in \mathbb{R}^{d \times d}$.

The parent-cancer-to-RNADisease map is frozen by preferred label before edge extraction: COADREAD maps to "colorectal cancer", UCEC to "endometrial cancer", SARC to "soft tissue sarcoma", BRCA to "breast cancer", and LGG to "glioma". Matching applies Unicode NFKC normalization, case folding, trimming, and internal-whitespace collapse, followed by exact equality to one RNADisease preferred label. A zero or multiple match makes the cohort ineligible; no synonym, ontology-parent, descendant, or post-result aggregation fallback is allowed.

Alternative-prior construction and gates. An alternative-prior sensitivity reconstructed the disease–gene graph from frozen DisGeNET and Open Targets releases [15, 16]. Publications, accessions, and evidence sources shared with RNADisease or the temporal validation set were removed before mapping. Failure to trace a retained edge excluded that edge. This evidence-record de-duplication removed traceable direct overlap but cannot establish statistical independence between curated databases. The alternative prior was treated as a source-reconstruction robustness gate, not as an independent replication experiment. It was neither pooled with RNADisease nor used to tune the main model.

For each non-UCEC cohort, the applicable master simultaneous lower bound was required to exceed zero. The median feature-rank Spearman correlation with the RNADisease analysis had to be at least 0.40. The two ordered top-20 feature lists also required Jaccard overlap of at least 0.40. The historically informed 0.015 bound is reported only as a sensitivity. UCEC received the same rank and overlap analysis but remained feasibility-only because its H02, H07, S02, and K02 bounds are structurally unavailable. Failure of any performance, global-rank, or leading-feature gate removed that cohort's knowledge-guidance interpretation. No UCEC inferential claim was available to remove or support.

For a training partition of n patients and requested mean degree γ, the binary patient graph contains exactly $E^\star = \min\{\binom{n}{2}, \mathrm{round}(n\gamma/2)\}$ unordered non-self edges. All pairs are ordered by decreasing cosine similarity and then, for an exact tie, by the lexicographically ordered pair of SHA-256 patient identifiers; the first $E^\star$ pairs are retained and the last retained pair defines (ϵ, h_ϵ), the similarity and tie-hash boundary. A zero-norm feature vector has cosine similarity zero. Self-loops are added only inside the GCN operator and are excluded from $E^\star$ and reported degree. An isolated training node therefore retains only its operator self-loop. For a held-out patient, an anchor edge is present when its cosine exceeds ϵ, or equals ϵ and its pair hash is no greater than h_ϵ; only training anchors are eligible, and neither the boundary nor the target mean degree is retuned. This rule fixes exact training density and a deterministic one-way transfer boundary.

Patient-graph construction sensitivity. The binary-threshold graph is the primary architecture and is not assumed to be optimal. Uniform message weights separate neighbourhood membership from edge magnitude. Cosine-weighted and mutual-kNN variants are evaluated at matched training mean degree. These variants use the same outer and inner folds, training-contained preprocessing, feature selection, and tuning budget as the primary construction. A sign reversal or absolute paired-effect change above 0.005 restricts the conclusion to the binary construction. A non-primary graph cannot retune, replace, or rescue the primary analysis.

GCNDC uses two graph-convolutional layers, ReLU after the first, inner-fold-selected width 32/64/128, dropout 0/0.2/0.5, the same learning-rate and weight-decay grids, a 300-epoch cap, patience 30, and class-weighted cross-entropy. For the frozen training graph, let $d_j^{\mathrm{tr}} = 1 + \sum_{i \neq j} B_{ji}$. For held-out node u, let b_{uj} follow the frozen (ϵ, h_ϵ) rule above and $d_u = 1 + \sum_j b_{uj}$. Its exact one-way update is $h_u^{(\ell+1)} = \sigma\Big[d_u^{-1} h_u^{(\ell)} W^{(\ell)} + \sum_j b_{uj} (d_u d_j^{\mathrm{tr}})^{-1/2} h_j^{(\ell,\mathrm{tr})} W^{(\ell)}\Big]$. Training degrees and states remain unchanged; no held-out-to-training or held-out-to-held-out message is allowed.

GCNDC optimization and stopping. For GCNDC and every declared patient-level GCN adapter, unless an official native implementation requires a named alternative, dropout is applied once after the first

ReLU and before the second graph-convolutional layer. There is no input or softmax-output dropout. Adam uses $\beta_1 = 0.9$, $\beta_2 = 0.999$, and $\epsilon = 10^{-8}$ with the selected learning rate and weight decay. Inverse-frequency class weights are normalized to mean one within the current training fold. The frozen inner primary metric is evaluated after every epoch. Improvement must exceed 10^{-6}, patience is exactly 30 consecutive nonimproving epochs, and the earliest best weights are restored. A method-specific deviation is admissible only when it follows a prespecified reproducible implementation rule. Otherwise, the trial is ineligible.

For each named transfer, the locked 30% internal-audit partition is excluded from the transfer-training object. Within the remaining 70% development partition, a public domain-separated identifier hash assigns 60% to the deployment core and reserves 40% for uncertainty calibration; a second hash divides the reserved pool into disjoint, approximately equal temperature-fit and conformal-threshold subsets. Patients are allocated within class. When a released non-singleton cluster is the independent unit, every split uses its hash, preserves it intact, stratifies only if clusters are class-pure, and retains realized class counts otherwise. An odd unit is assigned to the conformal subset by the frozen hash. Model selection, preprocessing, filtering, ranking, graph construction, class weights, and fitting use only the deployment core. The unique configuration maximizes the frozen inner-validation objective in that core, with ties within 10^{-12} resolved by frozen order. One SHA-256-derived seed fits one core model, and that exact predictor is never refitted after either calibration step.

The exact frozen core-trained predictor generates logits first for the temperature-fit subset. A scalar temperature is fitted on only those logits by multinomial negative log likelihood and then frozen; when cluster-level inference is primary, equally weighted cluster-specific mean losses replace patient weighting. Optimizer failure or an absent source class makes the row ineligible. The same predictor and temperature score the disjoint conformal-threshold subset. With patient units, its class-conditional scores are ordered individually; with cluster units, the maximum score in each cluster and class is the conformal unit. For $r_k = \lceil 0.9(n_k + 1) \rceil$, q_k is the r_kth ordered score if $r_k \leq n_k$ and $+\infty$ otherwise. Sets are $\Gamma(x) = \{k : 1 - \hat{p}_k(x) \leq q_k\}$. A finite 90% class-specific threshold requires at least nine independent conformal units; an infinite threshold retains that class in every set, is labelled uninformative, and fails the calibrated-uncertainty gate. The unchanged predictor, temperature, and thresholds are applied externally. External evaluation assesses empirical marginal and classwise coverage, mean set size, singleton rate, and empty-set rate without changing any object. No audit overlap, calibration-subset reuse, class pooling, out-of-fold surrogate, post-calibration refit, target recalibration, or distribution-shift guarantee is permitted.

Comparator implementation contract.

Display label	Retained native structure and search axes	Implemented comparison rule
Rhee-RN-GCN reimplementation	Two Chebyshev branches (published orders 10/2), 32 filters, batch normalization, ReLU, average pooling, 1024/512 fully connected layers; top-1000 relation edges and two 128-unit relation layers	Published structure reimplemented without endpoint or information-set substitution; interpreted descriptively
GEDFN-multiclass adapter	Graph-masked input layer, gene-width first hidden layer, then 64/16 units; ReLU; method-native dropout, learning-rate, and penalty axes	Replace only the published binary output with a K-class softmax; keep graph-source scope explicit
ERGCN-one-way-inductive adapter	Two-layer residual GCN, published 64-to-class structure, ReLU, softmax, and Pearson patient-graph threshold/mean-degree axis	Replace transductive full-sample construction with the frozen one-way rule; threshold selection is inner-fold only
GCNCC-multiclass adapter	64-dimensional gene embedding, Geometric-AP clustering, cluster ranking, forward selection, and logistic classifier	Fit the complete binary pipeline one-versus-rest within training data and softmax the K logits
GeSubNet-derived-score adapter	Patient-M, Graph-M, Infer-M, VQ-VAE/Neo-GNN dimensions, link split, decoder threshold, and stage-specific learning/epoch axes	Apply the declared graph coverage and neutral-missing rule after resolving implementation discrepancies before fitting
FullTx-EN	All technically retained release-level genes; multinomial elastic net $\alpha = 0/0.25/0.5/0.75/1$, $C = 10^{-4}$–100; balanced weights	No RNADisease, association intersection, graph, or supervised truncation; a convergence failure is ineligible
InfoMatch-EN	Association-eligible manifest shared with ImRIHF and matched GCNDC; multinomial elastic net $\alpha = 0/0.25/0.5/0.75/1$, $C = 10^{-4}$–100; balanced weights	Exhaust all 35 configurations; select by inner primary metric; normalized class probabilities; ties by frozen UTF-8 class order; no graph; a convergence failure is ineligible
FullTx-GCNDC	Unweighted GCNDC on the full-transcriptome candidate universe; training-only ANOVA rank and $k = 64/128/256/512$	No RNADisease or association-derived filter; otherwise identical one-way graph boundary and native GCN grid
Common audit	Same outer/inner folds, training-only boundary, trial and hardware-hour ceilings; manifest equality within each declared information set; published default is trial 0	No post-outcome configuration substitution or favourable-trial replacement

Retrospective analysis lock and prior-exposure disclosure

The analysis specification, including grids, thresholds, endpoints, cohort roles, exclusion rules, and known prior outcome exposure, was fixed before new outputs were inspected. This chronology limits post-output adaptation but does not restore outcome ignorance; analyses of previously public cohorts are therefore described as retrospective rather than prospective, confirmatory, or independently reproduced.

Execution separated association-object construction, patient-model fitting, prediction generation, label joining, and scoring. Association scores were fixed before patient modelling, and audit and external outcomes were not used during fitting or prediction generation. Predictions were fixed before they were joined to labels, and no stage was repeated after downstream outcome access. An execution failure remained failed, except for one identical predeclared infrastructure retry. This separation limits adaptive behaviour during execution but does not erase historical knowledge of earlier analyses.

Hypothesis and analysis key

The prespecified computational contract assigns the H/S/K/X coordinates to one permanently frozen 29-slot structure. H01–H11 are direct component or strict-common transfer contrasts. S01–S07 are internal full-transcriptome contrasts. K01–K05 compare the direct non-expression cancer-matched prior with the maximum over the prespecified generic-curation, wrong-disease, and technical-measurability controls. X01–X06 compare ImRIHF with FullTx-EN and FullTx-GCNDC in METABRIC, CGGA, and GSE14333, respectively. The design specifies one Holm adjustment and one fixed-dimension joint max-T object. The revision does not make an aggregate master-family passage claim. Table S4E retains all 29 identifiers and reports only the verified coordinate-level quantities used in the revised interpretation. H01 and H06 use READ-positive F1, H02–H05 and H07–H10 use unweighted macro-F1, and H11 uses rectum-positive F1 for the fixed 250-colon/39-rectum endpoint. H02, H07, S02, and K02 remain visible UCEC feasibility slots with raw $p = 1$ because the MSEAC audit contains ten cases.

UCEC cannot enter a cohort-level or multi-cohort inferential statement. Score-map, graph-null, transform, matched-marginal, source-reconstruction, topology, and transfer-negative-control analyses remain veto-only diagnostics. C01–C12 denote the fixed archived-candidate challenge genes.

Retrospective threshold provenance. Exact design thresholds were specified after public labels and earlier analysis results were already known. They are therefore interpreted as historically informed engineering sensitivities rather than outcome-blind forecasts, power priors, or confirmatory margins. Reported effects, marginal intervals, and raw probabilities are realized analysis outputs. Displayed values use consistent precision within each result family, while calculations used unrounded values. The displayed 0.010, 0.015, and 0.020 effect margins and the separately specified drift tolerances can only remove an interpretation. Under the prespecified contract, a master contrast requires a conservative master-Holm value below 0.05, a conservative master simultaneous lower bound strictly above zero, and no mapped veto. This revision makes no aggregate master-family passage statement. H02, H07, S02, and K02 remain gate-ineligible structural UCEC feasibility slots.

The primary-budget rule uses the first 100 configurations in each hashed method-native order or complete exhaustion of a smaller finite grid; the doubled-budget rule appends configurations 101–200 without changing any fold, preprocessing step, objective, search algorithm, or order. The prespecified resource rule applies a benchmark-fixed method-specific hardware-hour ceiling H_m to the primary budget and $2H_m$ to the doubled budget. InfoMatch-EN and FullTx-EN each exhaust their finite 35-configuration grid inside the primary budget, so the doubled row is labelled exhausted and receives no artificial trial. Matched GCNDC is the prespecified mechanism comparator, FullTx-GCNDC the prespecified full-transcriptome architecture control, and FullTx-EN the prespecified non-graph full-transcriptome control. In the descriptive repeated-CV budget sensitivity and COADREAD/UCEC/SARC/BRCA/LGG order, the observed primary/doubled ImRIHF–FullTx-GCNDC pairs are 0.487–0.462/0.489–0.464, 0.539–0.514/0.541–0.516, 0.814–0.793/0.816–0.795, 0.721–0.704/0.723–0.706, and 0.759–0.744/0.761–0.746. The prespecified robustness rule treats a reversal or absolute paired-gap drift above 0.005 as adverse.

Trial 0 is the published/native default. Before any inner-validation score is observed, all remaining configurations are ordered by the SHA-256 hash of a canonical configuration string under a method-domain-separated seed. Trial-count and hardware-hour views are separate, noninterchangeable audits, and the prespecified budget-robustness rule requires concordance in both.

Intermediate-fusion transformation and implementation checks. After matching expression and association features, let X_{ni} denote the normalized expression of mRNA i in patient n. Let $a_{ic} \in (0, 1)$ denote the locked LAGCN association score between mRNA i and the known parent cancer c. The index c is shared by the cohort and is not the unknown patient subtype. The implemented transformation is

$$\widetilde{X}_{ni}^{(c)} = 2X_{ni}a_{ic} \exp(-\delta|X_{ni}|) .$$

The value $a_{ic} = 0.5$ is only the algebraic reference at which the relevance multiplier $2a_{ic}$ equals one on the locked score scale. It is not a posterior-probability threshold. The separate negative-exponent term attenuates extreme magnitudes when $\delta > 0$. The full transformation therefore equals X_{ni} at $a_{ic} = 0.5$ only when $\delta = 0$. Unit tests at $\delta = 0$, $a_{ic} = 0.5$, $X_{ni} = 0$, and fixed nonzero examples reproduced this equation within 10^{-8}. Archived outputs produced by a different transformation were excluded.

Equation (7) is a robust separable prior transform, not a biological law. Its form was fixed for the locked retrospective analysis by four statistical constraints: preserve expression sign, map zero expression to zero, make association score 0.5 neutral under a linear nonnegative relevance factor, and bound the influence of extreme standardized expression with one nonnegative robustness parameter. Under these constraints, the simplest linear relevance factor is $2a_{ic}$, and the selected one-parameter redescending expression score is $X \exp(-\delta|X|)$. These constraints motivate but do not uniquely derive this choice.

The constraint-compatible transform family is frozen as exponential, rational monotone $X/(1+\delta|X|)$, and saturating monotone $\text{sign}(X)\{1-\exp(-\delta|X|)\}/\delta$, with its continuous $\delta = 0$ limit. The knowledge-guidance interpretation is restricted to settings in which all three transform-family effects are positive and their descriptive lower bounds remain above zero. For Equation (7), an outer-split proportion above 1% beyond the turning point $1/\delta$ fails the exponential-form mechanism interpretation. Covariate-matched score-map permutation and a separate continuous matched-marginal random-weight control test whether a positive paired F1 or macro-F1 difference depends on gene identity rather than the score distribution or measured gene-exchangeability covariates.

Theoretical alignment and bounded influence

Proposition 1 (alignment criterion for fixed $\delta \geq 0$). Define $Z_i^{(\delta)} = X_i \exp(-\delta|X_i|)$ and restrict the index set to retained features with $w_i(\delta) > 0$. For subtype k, let π_k be its prevalence, $\mu_{ki}^{(\delta)} = \mathbb{E}(Z_i^{(\delta)} \mid Y = k)$, $b_i(\delta) = \sum_k \pi_k\{\mu_{ki}^{(\delta)} - \mu_i^{(\delta)}\}^2$, and $w_i(\delta) = \sum_k \pi_k \operatorname{Var}(Z_i^{(\delta)} \mid Y = k)$. Define $m_i = (2a_{ic})^2$, $\omega_i(\delta) = w_i(\delta)/\sum_j w_j(\delta)$, and $r_i(\delta) = b_i(\delta)/w_i(\delta)$, and assume $\sum_i m_i w_i(\delta) > 0$; otherwise $R_\delta(M_c)$ is undefined. For the diagonal-scatter separability ratio

$$R_\delta(M_c) = \frac{\operatorname{tr}(M_c S_B^{(\delta)})}{\operatorname{tr}(M_c S_W^{(\delta)})} = \frac{\sum_i m_i b_i(\delta)}{\sum_i m_i w_i(\delta)},$$

we have

$$R_\delta(M_c) - R_\delta(I) = \frac{\operatorname{Cov}_{\omega(\delta)}\{m_i, r_i(\delta)\}}{\mathbb{E}_{\omega(\delta)}(m_i)}.$$

Indeed, $R_\delta(M_c) = \mathbb{E}_{\omega(\delta)}\{m r(\delta)\}/\mathbb{E}_{\omega(\delta)}(m)$ and $R_\delta(I) = \mathbb{E}_{\omega(\delta)}\{r(\delta)\}$; subtraction gives the identity. Thus, at any fixed damping value, the prior improves this surrogate if and only if squared gene weights are positively aligned with transformed feature-specific subtype signal-to-noise ratios. The result does not guarantee improved graph classification. For $\delta > 0$, $g_\delta(x) = x\exp(-\delta|x|)$ has derivative magnitude at most 1 and maximum absolute value $1/(e\delta)$, so it is 1-Lipschitz and bounded. Inner-fold selection fixes δ before outer-test transformation but does not convert the conditional identity into a performance guarantee.

Statistical analysis

Repeated 20×5 cross-validation and corrected-t summaries are descriptive. Each master coordinate instead uses one prediction pair per locked audit or external patient. The independent unit is one patient unless the frozen release manifest identifies repeated observations, sites, or sampling clusters that define dependence, in which case it is the entire released cluster. There is one controlling estimand, not an interchangeable finite-census and population pair. Let $S_m = 1$ denote eligibility under the exact release, one-primary-tumour, mapping, endpoint, and pre-outcome QC rules, and let $\mathscr{P}_m$ denote the source-specific cluster distribution represented by that release's accrual and measurement regime. Conditional on the frozen development data and fitted algorithms $\mathscr{A}_m$, the estimand is the empirically class-standardized paired contrast $\Delta_m^{\text{std}} = d_m\big[\sum_k \pi_{mk} E_{\mathscr{P}_m}\{z_{mi} \mid Y_i = k, S_m = 1, \mathscr{A}_m\}\big]$. Here $\pi_{mk} = N_{mk}/N_m$ is the observed class fraction in the complete eligible release frame and defines the primary finite-release estimand. Audit clusters must be independent and exchangeable within class with the eligible source frame. Because these are convenience accruals rather than probability samples, the working model supports only source- and release-conditional retrospective interpretation, never prevalence, geographic, endpoint-wide, or clinical-population generalization. If within-class exchangeability is not defensible, the output is a finite-manifest descriptive contrast with raw $p = 1$ and no confidence bound. Let n_{mk} be the evaluable audit count and $\bar{z}_{mk}$ its class mean; then $\widehat{\mu}_m = \sum_k \pi_{mk}\bar{z}_{mk}$ and $\widehat{\Delta}_m = d_m(\widehat{\mu}_m)$. A mandatory uncertainty sensitivity jointly resamples whole release-frame clusters

and class composition through both a nonparametric release-frame cluster bootstrap and a direct class-weight draw $\tilde{\pi}_m \sim \text{Dirichlet}(N_{m1} + 1/2, \dots, N_{mK_m} + 1/2)$ based only on complete release-frame counts; no multinomial count resampling is performed. A sign reversal or lower-bound drift above 0.005 in this sensitivity suppresses population-leaning wording and leaves only the fixed finite-release contrast. For $f(a, b, c) = 2a/(2a + b + c)$,

$$\nabla f(a, b, c) = \frac{[2(b + c), -2a, -2a]}{(2a + b + c)^2}.$$

The gradient ∇d_m combines the two methods' fixed metric terms: READ-positive F1 for COADREAD, rectum-positive F1 for GSE14333, and the fixed unweighted class terms for the multiclass coordinates.

For cluster g, define the paired influence contribution

$$S_{gm} = \sum_{i \in g} \frac{\pi_{m,Y_i}}{n_{m,Y_i}} \nabla d_m(\hat{\mu}_m)^\top (z_{mi} - \bar{z}_{m,Y_i}), \qquad \hat{s}_m^2 = \frac{G_m}{G_m - 1} \sum_{g=1}^{G_m} S_{gm}^2.$$

This preserves covariance between algorithms and allows arbitrary within-cluster dependence while requiring independence between frozen clusters. For every pre-output-qualified and computable master coordinate, $B = 199{,}999$ fixed-seed Rademacher multipliers give $T_m = \hat{\Delta}_m / \hat{s}_m$ and $T^*_{mb} = \sum_g \xi_{gb} S_{gm} / \hat{s}_m$. Coordinates sharing patients use the same multipliers, and different cohorts use independent streams. The two-sided weak-null compatibility probability is $p_m = \{1 + \sum_{b=1}^{B} I(|T^*_{mb}| \geq |T_m|)\}/(B + 1)$. It is asymptotic cluster-robust compatibility analysis for $H_{0m} : \Delta_m^{\text{std}} = 0$, conditional on frozen algorithms, fixed empirical class standardization, and the stated source-exchangeability model. It is neither a finite-sample randomization test nor uncertainty over retraining. Historical access to cohorts, labels, architecture, and earlier results means that every probability and adjustment remains retrospective and descriptive, irrespective of magnitude.

The primary statistical engine is a paired whole-cluster influence-function studentized Rademacher multiplier bootstrap; all weak-null and max-T calculations below use that same engine.

The master family always contains exactly 29 Holm slots, and the max-T dimension cannot be selected from outcomes. Let the pre-output coordinate set $\mathscr{E}_0 \subseteq \{1, \dots, 29\}$ be frozen before any prediction is generated or evaluation label is joined, using only exact release identity, endpoint and cluster manifests, annotation-only feature representability, fixed class-count gates, a defensible source-exchangeability declaration, and the prespecified simulation qualification. Observed metric denominators, effect direction or magnitude, standard errors, influence values, dominant-cluster shares, and multiplier tails cannot add or remove a coordinate. The ten-MSEAC design excludes H02, H07, S02, and K02 from $\mathscr{E}_0$; each occupies its master slot with raw and master-adjusted $p = 1$ and no bound. Every other pre-output-excluded row likewise receives a zero multiplier coordinate, raw $p = 1$, deterministic raw-tail envelope $[1, 1]$, and an NA bound. A scheduler or I/O interruption may be retried once only under identical conditions; numerical nonconvergence cannot be repaired with another seed or setting. Holm is applied once to all 29 fixed probabilities. It controls this declared retrospective claim family conditionally but cannot undo historical exposure or establish prospective confirmation.

Every computable $m \in \mathscr{E}_0$ remains in the maximum on every draw, including a row whose claim is later vetoed by a quality diagnostic. The fixed-dimension draws are $M_b = \max\{0, \max_{m \in \mathscr{E}_0} T^*_{mb}\}$. If any coordinate in $\mathscr{E}_0$ is noncomputable because a prediction, metric denominator, influence value, or standard error is missing or non-finite, the joint max-T object and all joint lower bounds are globally NA. The dimension is never reduced to rescue surviving rows. A computable row with $G_m < 30$ or squared-influence share $S_{gm}^2 / \sum_{g'} S_{g'm}^2 > 0.20$ remains in M_b, but its raw probability is set to 1, its bound is suppressed, and its claim fails. When $\mathscr{E}_0$ is nonempty and every coordinate is computable, $c_{0.95} = Q_{0.95}(M_b)$ and $L_m = \hat{\Delta}_m - c_{0.95}\hat{s}_m$ form one asymptotic simultaneous one-sided 95% lower-confidence set over the fixed pre-output master family. The prespecified boundary is zero: a coordinate satisfies the master rule only if its conservative master-Holm value is below 0.05, its conservative joint lower bound strictly exceeds zero, and every mapped quality gate passes. These historically informed

retrospective analyses are not used to claim confirmatory passage; the 0.010/0.015/0.020 margins remain descriptive sensitivities only.

This is the joint one-sided max-T confidence construction.

The noninferential extensions use fixed sentinels: a fixed-finite-census row and an ineligible-comparison-universe row each retain the occupied master slot with raw and master-adjusted $p = 1$ and joint lower bound NA.

The fixed thirty-object familywise 95% Monte Carlo envelope contains 29 raw-tail slots plus one fixed-dimension master max-T quantile. Every computable $m \in \mathscr{E}_0$ receives an equal-tailed exact Clopper–Pearson interval with confidence $1 - 0.05/30$, or $0.05/60$ per side, transformed through the plus-one rule $[\ell_m, u_m] \mapsto [(1 + B\ell_m)/(B + 1), (1 + Bu_m)/(B + 1)]$. A pre-output-excluded or post-output-vetoed raw slot remains conservatively $[1, 1]$ for master Holm without removing a computable coordinate from M_b. For ordered fixed-dimension draws $M_{(1)} \leq \cdots \leq M_{(B)}$, $B = 199{,}999$ and the 0.95 quantile use exact-binomial ranks $r_L = 189{,}691$ and $r_U = 190{,}305$; $M_{(r_U)}$ defines $L_m^- = \widehat{\Delta}_m - M_{(r_U)}\hat{s}_m$ only when the complete $\mathscr{E}_0$ object exists. A noncomputable coordinate makes the quantile and every L_m^- NA rather than triggering a smaller maximum. This thirty-object allocation audits Monte Carlo error, not selection. A separate whole-unit method-label-swap analysis may probe the stronger sharp null of method-label exchangeability, but assignment was not randomized. The swap result is descriptive and cannot create, rescue, or invalidate a master weak-null description.

The fixed interpretation map can remove but never add interpretations. A cohort-specific component statement requires its H coordinate; full-transcriptome competitiveness additionally requires both applicable FullTx coordinates; and disease-specific knowledge-guidance language additionally requires K. Every such statement also requires all deterministic vetoes mapped to it. Transform, matched-marginal, graph-construction, source-reconstruction, curation, and technical-measurability gates apply to all five internal analyses; the score-map veto applies to COADREAD, UCEC, and SARC, whereas degree-preserving graph rewiring applies to BRCA and LGG. H09 METABRIC additionally maps to BRCA H04 and X01–X02. H10 CGGA maps to LGG H05 and X03–X04. H11 GSE14333 maps to COADREAD H01 and X05–X06. All H/S/K/X coordinates share the master Holm and max-T objects; no coordinate or alpha can rescue another. Candidate, GeSubNet-adapter, and post-cutoff evidence cannot rescue prediction. UCEC is always reported as feasibility and is excluded from multi-cohort inference. GSE21050 and GSE17025 remain descriptive.

The 1,999 whole-pipeline patient-label permutations serve only as a global no-signal stress test of the descriptive learning workflow. They destroy label–feature association and therefore do not test equality between frozen algorithms, do not supply a master probability, and cannot rescue a coordinate. The whole-procedure simulation was designed to exercise the complete 29-slot master-family, interpretation-dependency, and veto program as one object. Its prespecified grid included the global null, least-favourable partial-null configurations, missing fits, ineligible comparators, dominant clusters, and zero or near-zero metric denominators. This revision does not use a realized coverage, false-interpretation, power, or simulation-passage claim. H02, H07, S02, and K02 remain adverse descriptive slots regardless of simulation because the locked audit contains only ten independent MSEAC units.

Covariate-matched score-map and continuous-weight controls. For each of COADREAD, UCEC, and SARC, 999 cancer-specific score-map randomization pipelines permute the complete locked a_{ic} vector without replacement only inside joint gene-covariate strata. At each fitting boundary, strata are constructed from prior degree decile, pre-cutoff evidence-record-count tertile, pre-cutoff publication-count tertile, current-training expression-variance quartile, current-training missingness category (0, > 0–1%, or > 1–5%), current-training detection-rate bin, gene-length and GC-content bins, uniquely mappable-fraction bin, frozen unique-probe and ambiguous-mapping status, and the frozen count of named external platforms on which the gene is representable. Unavailable technical fields form an explicit missing category and are never inferred from outcomes. These variables and cut points are computed before any

outcome is used.

Cells with fewer than eight eligible genes are merged deterministically with the nearest cell along the frozen axis order: missingness, detection, variance, mappability, probe status, publication count, evidence count, degree, and platform count. Equal-distance ties are resolved toward the lower bin. The resulting map is fixed before fitting. This is a prespecified diagnostic reference, not an unrestricted gene-exchangeability assumption or a null hypothesis in the 29-slot computational master family.

Randomization index b denotes one complete pipeline replicate, not one gene mapping reused across folds. At every fitting boundary h, strata and merges are recomputed from that boundary's training data, and the hash of (b, h) fixes the boundary-specific permutation. Thus the same b coherently indexes the complete leakage-safe set of fold-specific maps. The real map and every randomized replicate undergo the complete 20×5 nested pipeline. This includes training-contained feature selection, independent inner-fold selection of δ and graph hyperparameters from the same frozen grids, graph construction, fitting, and outer prediction. Splits and optimization seeds are paired across maps.

With T_{real} denoting the real-map mean outer-fold metric and T_b its counterpart for replicate b,

$$p_{\text{score}} = \{1 + \sum_{b=1}^{999} I(T_b \geq T_{\text{real}})\}/1000.$$

The corrected interval instead uses each outer fold's real-map metric minus its median matched-permutation metric and quantifies split sensitivity. The nominal diagnostic probability describes the chosen score-reference ensemble conditional on measured gene covariates. It does not establish gene-score exchangeability, cannot enter Holm adjustment, and is not a patient-level efficacy test.

A separate continuous random-weight control uses 999 complete pipeline replicates sampled within the same merged strata while matching the first four score moments. It is a veto-only robustness check outside the 29-slot computational master family. A knowledge-guidance interpretation requires the real-score effect to remain positive and to drift by no more than 0.005 relative to its prespecified diagnostic contrast. This control cannot rescue a failed global gate.

For the Figure 4 factorial diagnostic, damping absent is $\delta = 0$ and damping present is δ_+ selected independently inside each inner loop from the strictly positive frozen grid. The primary model still searches the original grid including zero; a primary zero selection is reported as zero and is never relabelled as damping present.

Prior perturbation and graph-null controls

Prior-edge deletion and degree-matched addition are evaluated separately at 5%, 10%, 20%, 30%, and 50% with 50 fixed seeds in each of COADREAD, UCEC, SARC, BRCA, and LGG; seeds 51–100 are automatically executed where the frozen interval-width rule is triggered. At 20%, the observed intact; delete/add; matched-null delete/add; topology-advantage delete/add; and median top-20 Jaccard delete/add results are COADREAD 0.487; 0.478/0.473; 0.462/0.461; 0.016/0.012; 0.57/0.46; UCEC 0.539; 0.529/0.523; 0.517/0.515; 0.012/0.008; 0.54/0.43; SARC 0.814; 0.805/0.800; 0.798/0.794; 0.007/0.006; 0.60/0.48; BRCA 0.721; 0.716/0.708; 0.703/0.700; 0.013/0.008; 0.57/0.46; and LGG 0.759; 0.750/0.744; 0.738/0.736; 0.012/0.008; 0.54/0.46. These completed perturbation analyses are descriptive vetoes and do not create a robustness claim.

Degree-preserving graph rewiring. Degree-preserving graph rewiring is a prespecified topology robustness reference rather than a test in the 29-slot computational master family. Each binary training-patient graph is rewired with a symmetric lazy Maslov–Sneppen transition that preserves its degree sequence and edge count. Starting from the observed product state, the Besag–Clifford parallel construction

uses $100E^{\star}$ attempted transitions per component to a common hub. A total of 999 independently seeded branches then generates complete null-pipeline statistics.

The nominal rank

$$p_{\text{graph}} = \{1 + \sum_{b=1}^{999} I(T_b \geq T_{\text{obs}})\}/1000$$

is reported only as a topology-reference diagnostic. It does not enter the 29-slot computational master family or its Holm adjustment. Any failed branch remains in the denominator and counts adversely. Association LAGCN embeddings, a_{ic}, Equation (7) weights, preprocessing, patient features, and folds remain fixed. The observed state and every branch rerun the identical training-contained patient-graph tuning, classifier fitting, and one-way held-out prediction protocol. This diagnostic isolates patient-graph topology, while alternative-prior analyses separately perturb the prior.

A source-reconstruction prior from frozen DisGeNET and Open Targets inputs removes traceable shared evidence with RNADisease but cannot establish database independence. In COADREAD/UCEC/SARC/BRCA/LGG order, its five-cohort differences are +0.031/+0.027/+0.024/+0.028/+0.030, descriptive sensitivity lower bounds outside the master family are 0.020/0.018/0.017/0.017/0.020, and median feature-rank Spearman correlations are 0.47/0.45/0.43/0.44/0.49. The observed top-20 intersection/union/Jaccard values are 13/27/0.481, 12/28/0.429, 12/28/0.429, 13/27/0.481, and 14/26/0.538. These fractions are exact consequences of two 20-gene lists. These gates remain veto-only outside the computational master family.

In descriptive repeated-CV robustness analyses for COADREAD/UCEC/SARC/BRCA/LGG, exponential-transform effects are 0.037/0.029/0.024/0.029/0.032; rational effects are 0.035/0.028/0.023/0.027/0.030 with descriptive non-master lower bounds 0.0190/0.0160/0.0153/0.0170/0.0200; and saturating effects are 0.034/0.027/0.022/0.026/0.029 with descriptive non-master lower bounds 0.0180/0.0152/0.0151/0.0160/0.0190. Median/maximum proportions beyond the selected turning point are 0.1/0.4%, 0.2/0.6%, 0.2/0.5%, 0.1/0.3%, and 0.1/0.4%. Matched-marginal score effects are 0.028/0.020/0.016/0.024/0.027 with descriptive non-master lower bounds 0.0154/0.0110/0.0103/0.0160/0.0190. All displayed lower bounds in these three five-cohort sequences are rounded to four decimal places; decisions use unrounded values. Binary, cosine-weighted, and mutual-kNN graph effects are 0.037/0.029/0.024/0.029/0.032, 0.035/0.027/0.022/0.027/0.030, and 0.033/0.026/0.021/0.025/0.028; each stays within 0.005 of the primary effect.

The headline direct non-expression cancer-matched prior excludes expression-only, prognostic, subtype-outcome, evaluated-cohort, and overlapping evidence-source records; the broader RNADisease graph is historical sensitivity only. The K control set is frozen before outputs and contains exactly five prespecified controls: gene degree, evidence-record count, cutoff-date publication count, a degree-and-evidence-matched wrong-disease prior, and a technical-measurability-only score. The technical score freezes detection, missingness, variance, length, GC, mappability, probe, and platform fields without outcome imputation. For each cohort, K_c is the paired difference between the headline-prior effect and the maximum effect among this fixed control set; the control maximum is recomputed inside every paired whole-cluster multiplier draw so that the raw probability and lower bound account for selection of the strongest control. K01–K05 are permanently occupied coordinates inside the 29-slot master family, not a separate Holm-5 family. In COADREAD/UCEC/SARC/BRCA/LGG order, the observed headline effects are +0.029/+0.025/+0.021/+0.026/+0.029, the strongest-control effects are +0.006/+0.005/+0.004/+0.005/+0.005, and the gaps are 0.023/0.020/0.017/0.021/0.024. Raw probabilities for K01/K03/K04/K05 are 0.0010/0.0060/0.0020/0.0012. Separate Holm-5 and five-coordinate lower-bound calculations are not used. K02 instead has fixed raw $p = 1$, master-adjusted $p = 1$, and lower bound NA under the ten-MSEAC feasibility design. The 0.010 values are retained only as a historical engineering sensitivity; the prespecified master rule uses the zero boundary. A K coordinate cannot create or rescue performance evidence. The K results are interpreted descriptively within this retrospective study. Association validation groups edges by publication and evidence source and reports

temporal, source-disjoint, and entity-disjoint performance rather than treating random-edge prediction as independent generalization.

External transfer and candidate-expression analysis

The frozen transfer bundle implements a single-sample algorithm rather than cohort-level batch correction. Its order is exact. First, the documented release scale is retained for $\log_2$-normalized data, while other nonnegative values receive $\log_2(x + 1)$. Second, a versioned platform annotation maps probes to version-stripped Ensembl genes; multi-gene probes are removed and multiple valid probes for one gene are collapsed by within-sample median. Third, annotation availability alone, before any external expression value, label, or prediction is inspected, defines the evaluation anchor universe $\mathcal{U}$ as the intersection of the development technical-pass genes and genes representable under the declared comparison scope. The development refit uses that same $\mathcal{U}$. Fourth, all observed genes in $\mathcal{U}$ receive within-sample midranks and normal scores $z_{ig} = \Phi^{-1}\{(r_{ig} - 0.5)/p_i^{\text{obs}}\}$, where p_i^{obs} counts the observed anchor genes; this rank transform is never recomputed on a selected-feature list. Fifth, only after anchor-universe scoring are the frozen selected features extracted. A sample with more than 5% absent required selected genes is ineligible; otherwise each absent selected z_{ig} is filled with that gene's frozen development pre-standardization mean μ_g. Sixth, every selected entry is standardized as $x_{ig} = (z_{ig} - \mu_g)/\sigma_g$ using frozen development μ_g and σ_g; genes with zero development σ_g were already excluded. Equation (7), the patient graph, calibration, and prediction then receive this standardized vector. Missingness therefore maps to standardized zero without assuming that the raw or normal-score value itself is zero. The algorithm estimates no target-cohort location, scale, quantile distribution, batch effect, or outcome-informed parameter and never substitutes a gene.

A second no-target-fit harmonization path replaces $\Phi^{-1}(u)$ with a development-frozen empirical reference quantile constructed only from mapped development expression, and every method is refitted in development under that transform. The feature universe, patient denominator, graph rule, tuning budget, calibration protocol, and prediction seal are unchanged; no target-cohort location, scale, quantile, batch, or outcome is estimated. This sensitivity cannot rescue the primary analysis. A cross-platform-robustness statement requires a positive paired effect under both transforms and absolute effect drift no greater than 0.010; otherwise the conclusion is restricted to the named cohort under the primary transform. The alternative-harmonization sensitivity was completed alongside the primary analysis.

A 999-branch cross-platform negative control was specified for both no-target-fit harmonization paths. Before each branch, a hash fixed the cohort, method, transform, replicate identifier, per-gene patient-permutation map, class-count-preserving label rotation, chance-envelope construction, numerical bounds, and the 0.005 gap threshold. Within each branch, every mapped gene is independently permuted across external patients under its frozen hash, preserving that platform's gene-wise marginal values and missingness pattern; the endpoint vector is independently rotated under a non-identity hashed permutation that preserves the exact class counts. Each branch traverses the unchanged annotation map, anchor rule, primary or alternative single-sample transform, missing-gene rule, frozen standardization, patient graph, calibration, prediction-set, and sealed prediction workflow, with no target-derived fit. This preserves platform and gene marginals while destroying patient-level expression–outcome alignment. The diagnostic is veto-only, remains outside the 29-slot master family, and cannot create or rescue positive transfer evidence. No result-level negative-control passage statement is used in this revision.

Every specified external analysis is retrospective and is governed by the pre-analysis lock. Public labels and class counts may have been historically known, so neither "untouched" nor outcome-naive is claimed. Before computation, the deployment-core predictor, feature universe, platform map, single-sample transforms, calibration objects, exclusions, and subgroups were frozen. Predictions were sealed before labels were joined. Each external patient connects only to development anchors; target-to-target edges are excluded. H09–H11 compare ImRIHF with matched GCNDC on strict-common graph coverage in METABRIC, CGGA, and GSE14333. X01–X06 compare ImRIHF with FullTx-EN and FullTx-

GCNDC on their native universes. The observed coordinate-level effects remain visible, but no aggregate competitive-transfer passage statement is made. GSE21050 and GSE17025 remain descriptive.

Before scoring, a covariate-availability manifest freezes every reliably released field for source site, specimen source or preservation, tumour purity, stage, age, sex, and ancestry or race. After predictions are sealed, performance, paired difference, minimum recall, and calibration are evaluated for every prespecified stratum with at least 30 patients and at least five patients per class. Smaller or unavailable strata support no subgroup claim. No missing covariate is imputed from outcomes and no stratum is selected by performance. A direction reversal suppresses a subgroup-robustness statement but cannot generate a new claim. Applicable secondary-use ethics, non-human-subjects, exemption, or data-use determinations were documented for each cohort.

External scopes are reported in separate rows and may not be pooled. The native-universe row applies each method's frozen development information set with its declared anchor and missingness rule. The portability row refits each method on its own development universe intersected with development and external platform annotations fixed without external expression values or labels. The strict-common graph row is narrower and is shared only by ImRIHF and matched GCNDC: one annotation-only $\mathscr{U}$ representable by both graph pipelines is frozen, and both methods are refitted in development on that identical anchor; fewer than 64 genes makes the row ineligible. FullTx-GCNDC and FullTx-EN remain prespecified controls on the same patients but retain separately labelled native and portability anchors and denominators. Neither is described as a strict-common model or enters H09–H11. Their native-universe paired contrasts enter X01–X06 and cannot be replaced by coverage-restricted or portability rows. H09–H11 use only the ImRIHF-versus-matched-GCNDC strict-common contrast, and GSE21050 uses the same two-method scope only for a descriptive endpoint-mapping sensitivity. No native or portability row can substitute for a failed strict-common graph gate. The displayed 256-feature mapping fractions are separate ImRIHF mapping checks and do not define $\mathscr{U}$.

The official 310-sample GSE21050 record contains untreated primary sarcomas: cohort 1 has 44 DDLPS, 51 LMS, 71 undifferentiated, 16 "Other", and one missing diagnosis, and cohort 2 has 18 DDLPS, 33 LMS, 65 undifferentiated, and 11 "Other". The endpoint-mappable set is 282; the 27 "Other" samples enter only label-coarsening sensitivity, and the missing-diagnosis sample is always excluded. Source-specific metrics and the interaction interval were evaluated descriptively.

Before predictions were joined to labels, the feature map, preprocessing, anchor graph, model, calibration, conformal thresholds, manifest, exclusions, and predictions were fixed. This boundary limits post-prediction adaptation but does not establish prior label ignorance or independent replication.

The named public cohorts provide retrospective, source-conditional evidence only and do not establish prospective confirmation or endpoint-wide generalization.

The historically selected 12-gene challenge subset comprises ITGB4, DPP7, PGAM1, TRAPPC6A, RPL35A, IBSP, RHOC, DEK, LY6E, RALA, BAG3, and MCM6; it was drawn from, but does not exhaust, the submitted candidate lists. The subset and directed contrasts were fixed before the external-expression audit, but the original candidate and subset selection was outcome-exposed and is not treated as systematic, independent, or suitable for estimating a panel-wide yield. All unmapped, discordant, and nonsignificant rows remain. This candidate-expression audit is separate from the predictive-transfer mapping above. Three fixed pairwise contrasts were used: GSE14333 READ (Rectum) versus COAD (Colon), 39 versus 250, with one record lacking a location label excluded; GSE17025 EEA versus SEA, 79 versus 12; and GSE21050 LMS versus DDLPS, 84 versus 62, with undifferentiated, "Other", and missing-diagnosis samples excluded from this statistic. When more than one eligible single-gene GPL570 probe represented a candidate, the probe with the largest median expression across all eligible samples in that cohort was selected without using class labels. This outcome-blind, external-distribution-dependent single-probe rule is distinct from the annotation-defined within-sample median collapse used for predictive transfer. Supplementary Figure S6c displays every eligible patient's processed-expression value for all 11 mapped genes; for visual comparability only, values are standardized within gene over the

eligible comparison samples, while all inferential calculations use the original processed-expression values. Panel d reports directed Hedges g and its pooled-SD approximate 95% interval. Two-sided Welch p values are BH-adjusted across all 12 fixed rows using full precision. These statistics use different variance estimators, and BH q, rather than whether the Hedges-g interval includes zero, defines significance. MCM6 had no eligible GPL570 probe and was retained as platform-unmapped with $p = q = 1$. Table S7A summarizes aggregate mapping, direction-concordance, and BH-significance accounting for the fixed subset and names the subsets meeting the BH-significance and joint direction-concordance/BH-significance criteria. This retrospective external-expression analysis is descriptive and does not establish causality, mechanistic validation, independent validation, or de novo discovery.

"Interpretable" denotes the transparent cancer-conditioned metric, audited feature weights, and reconstructable prediction traces; it does not imply biomarker stability, causality, clinical utility, or mechanistic proof.

Evidence status: the external-transfer and archived-candidate computational analyses were completed. The counts, estimates, intervals, probabilities, panels, and table rows are observed computational outputs generated under the stated retrospective safeguards. No result is used to claim prospective confirmation, causality, or de novo biomarker discovery.

Supplementary Tables

Supplementary Table S1A. Evidence register.

Evidence layer	Reported result	Integrity condition
Association source/edge analysis	Source-first denominator and performance from the complete eligible pre-cutoff RNADisease record; 9,182 edges and 4,928 mRNAs are historical archived-analysis counts only	Map independently of archived expression membership; intersect with each training-only anchor $\mathscr{U}$; mask before both kernels
Temporal holdout	90/414 post-cutoff positives; table 50/100/40/224; OR 2.80; 95% CI 1.74–4.52; BH $q = 0.012$	Zero training/validation-edge overlap
Computational master-family contract	H01–H11, S01–S07, K01–K05, and X01–X06 define 29 permanently occupied coordinates	One Holm adjustment across all 29 raw probabilities and one fixed-dimension master max-T object; no family-specific alpha or coordinate deletion
Internal master-coordinate interpretation	Point estimates, probabilities, and intervals are retrospective	H02/H07/S02/K02 are adverse feasibility slots by design; no confirmatory interpretation
Full-transcriptome scope	Locked-audit FullTx-EN 0.445/0.524/0.775; FullTx-GCNDC 0.448/0.526/0.777	Both FullTx controls are mandatory claim limiters; the corresponding H/S/X coordinates enter the master family; no result-selected comparator or subset-specific multiplicity family
GeSubNet-derived-score adapter	Scores 0.468/0.523/0.806; ImRIHF differences 0.019/0.016/0.008	Graph-coverage-aware derived score; exploratory BH 0.012/0.024/0.090; SARC interval crosses zero
Mechanism score identity	Real vs median of 999 complete covariate-matched score-map pipelines: 0.029/0.021/0.017	Veto-only nominal diagnostics 0.002/0.007/0.004; no master-family or master-Holm label
Internal molecular tasks	BRCA 0.729 vs 0.697; LGG 0.744 vs 0.709; H04/H05 differences 0.032/0.035	Direct audit-patient comparisons; PAM50 is expression-derived; graph rewiring is a separate veto
Alternative prior	Five-cohort DisGeNET/Open Targets deltas 0.031/0.027/0.024/0.028/0.030	Source-disjoint reconstruction, not independent replication; descriptive non-master lower bounds 0.020/0.018/0.017/0.017/0.020; rank Spearman 0.47/0.45/0.43/0.44/0.49; top-20 intersections/unions 13/27, 12/28, 12/28, 13/27, and 14/26, giving Jaccard 0.481/0.429/0.429/0.481/0.538
Disease-specificity coordinates	K01–K05 observed headline-prior gaps over the strongest fixed curation/wrong-disease/technical control: 0.023/0.020/0.017/0.021/0.024	K enters the 29-slot master family; separate Holm-5 and five-coordinate lower bounds are not used; the historical 0.010 margin is sensitivity-only
External transfer	Completed source-conditional retrospective evaluations in METABRIC, CGGA, and GSE14333	H09–H11 use strict-common matched GCNDC; X01–X06 use FullTx-EN and FullTx-GCNDC native universes. Coordinate-level effects are reported without an aggregate competitive-transfer passage statement
Cross-platform negative control	999 pre-hashed gene-wise patient permutations plus exact-class-count label rotations under both no-target-fit transforms	Preserves platform/gene marginals while breaking patient signal; veto-only and outside the master family. No result-level passage claim is made
Endpoint/sensitivity transfers	GSE14333 is fixed H11; GSE21050 remains descriptive	Rectum-positive F1 is immutable for H11; GSE21050 stays outside alpha control; both transfer analyses were completed under their declared scopes
Historical challenge subset	All 12 revision-stage historical identities remain fixed challenge rows; the subset does not exhaust the submitted candidate universe, and Table S7B reports mapping, BH-significance, joint-criterion status, and interpretation for every gene	All mapped, unmapped, concordant, discordant, significant, and nonsignificant rows remain in the denominator; no panel-yield or de novo-discovery claim
Calibration/small classes	Label permutation is a no-signal diagnostic only; 60/40 deployment-core/uncertainty split within development; disjoint temperature/conformal halves; class-conditional 90% sets	Exact same predictor produces all logits; finite threshold requires at least nine independent conformal units; no exact shift-coverage claim
Supplementary Table S7C	Literature context only	No independent-validation or mechanistic-evidence credit

Supplementary Table S1A (continued). Dependence-aware association validation.

Partitioning rule	Positive/matched-unknown pairs	Dependence groups	Metric	ImRIHF	NMCMDA	Δ	Cluster 95% CI
Evidence-source-disjoint	2,184/2,184	14 source groups	AUROC	0.918	0.889	0.029	0.017–0.041
Evidence-source-disjoint	2,184/2,184	14 source groups	AUPR	0.902	0.864	0.038	0.022–0.054
Publication-disjoint	3,072/3,072	1,146 publication groups	AUROC	–	–	0.030	0.019–0.041
Publication-disjoint	3,072/3,072	1,146 publication groups	AUPR	–	–	0.039	0.025–0.053
Entity-disjoint	1,536/1,536	384 entity groups	AUROC	–	–	0.028	0.012–0.044
Entity-disjoint	1,536/1,536	384 entity groups	AUPR	–	–	0.036	0.018–0.054

The evidence-source-, publication-, and entity-disjoint analyses group association edges by the stated dependence unit rather than treating random-edge prediction as independent generalization. A dash indicates that the corresponding absolute method value was not reported for that row; no value is reconstructed from the paired difference.

Supplementary Table S1B. Comparator coverage and search-budget summary.

Panel	Displayed values and declared fields	Search-budget rule	Scope
Native universe	Eligibility, graph coverage, missingness, metrics, and convergence determine each comparison	First 100 frozen configurations or smaller-grid exhaustion; doubled rule appends 101–200	Method-specific information set remains explicit
Portability	External analyses use the development–platform intersection defined separately for each method	Same frozen configuration order; no external value or label defines the intersection	External numerical results remain descriptive
Strict common	One annotation-only anchor $\mathcal{U}$ shared only by ImRIHF and matched GCNDC; ineligible below 64 genes	Same patient rows, folds, frozen order, and budget rules for those two graph pipelines	H09–H11 map to this contrast; X01–X06 map to FullTx-GCNDC/FullTx-EN native-universe contrasts
Primary/doubled repeated-CV sensitivity	Prespecified FullTx-GCNDC. In COADREAD/UCEC/SARC/BRCA/LGG order, primary pairs: 0.487–0.462, 0.539–0.514, 0.814–0.793, 0.721–0.704, 0.759–0.744	In the same cohort order, doubled pairs: 0.489–0.464, 0.541–0.516, 0.816–0.795, 0.723–0.706, 0.761–0.746	Completed with no post-outcome comparator selection

Trial budgets were applied identically within each declared method-specific search space; no hardware-efficiency or economic claim is made.

Supplementary Table S1C. Final patient denominators.

Cohort	Frozen endpoint labels	Inclusion/exclusion accounting	Final sample counts
COADREAD	COAD/READ project labels; READ positive	Locked 332; release-mapped/unique-primary/QC-pass 332/332/332; duplicate/non-primary/missing-label/QC exclusions 0/0/0/0	Final 254/78 (n=332)
UCEC	EEA/MSEAC/SEA; no post-result regrouping	Locked 430; release-mapped/unique-primary/QC-pass 430/430/430; duplicate/non-primary/unmapped-or-missing/QC exclusions 0/0/0/0	Final 311/21/98 (n=430)
SARC	DDLPS/LMS/frozen enumerated pooled-other list	Locked 257; release-mapped/unique-primary/QC-pass 257/257/257; duplicate/non-primary/unmapped-or-missing/QC exclusions 0/0/0/0	Final 58/103/96 (n=257)
BRCA	Basal-like/HER2-enriched/Luminal A/Luminal B; Normal-like excluded	PAM50 label source fixed; PAM50 predictors blocked; final 190/82/562/202	Final total 1,036
LGG	IDH-wildtype/IDH-mutant non-codeleted/IDH-mutant codeleted	DNA-defined label source; defining genes blocked from candidate evidence; final 90/249/172	Final total 511

The denominator audit reconciled the locked-manifest total, release-mapped identifiers, unique participants, primary-tumour expression records, release-level quality control, exclusions, and final class counts. All tabulated numbers are the final analyzed denominators.

Supplementary Table S1D. Five-cohort 0/5/10/20/30/50% prior-perturbation summary.

Cohort	Series	0%	5%	10%	20%	30%	50%
COADREAD	Perf-D	0.487	0.485	0.483	0.478	0.467	0.445
COADREAD	Perf-A	0.487	0.483	0.480	0.473	0.461	0.437
COADREAD	Null-D	0.456	0.458	0.459	0.462	0.460	0.443
COADREAD	Null-A	0.456	0.457	0.458	0.461	0.455	0.435
COADREAD	Adv.-D	0.031	0.027	0.024	0.016	0.007	0.002
COADREAD	Adv.-A	0.031	0.026	0.022	0.012	0.006	0.002
COADREAD	Jacc.-D	1.00	0.91	0.81	0.57	0.39	0.16
COADREAD	Jacc.-A	1.00	0.86	0.71	0.46	0.28	0.09
UCEC	Perf-D	0.539	0.537	0.535	0.529	0.516	0.493
UCEC	Perf-A	0.539	0.535	0.531	0.523	0.510	0.486
UCEC	Null-D	0.515	0.516	0.517	0.517	0.510	0.491
UCEC	Null-A	0.515	0.516	0.516	0.515	0.504	0.484
UCEC	Adv.-D	0.024	0.021	0.018	0.012	0.006	0.002
UCEC	Adv.-A	0.024	0.019	0.015	0.008	0.006	0.002
UCEC	Jacc.-D	1.00	0.90	0.78	0.54	0.37	0.15
UCEC	Jacc.-A	1.00	0.84	0.67	0.43	0.26	0.08
SARC	Perf-D	0.814	0.813	0.811	0.805	0.793	0.769
SARC	Perf-A	0.814	0.811	0.808	0.800	0.789	0.763
SARC	Null-D	0.795	0.797	0.798	0.798	0.787	0.767
SARC	Null-A	0.795	0.796	0.797	0.794	0.783	0.761
SARC	Adv.-D	0.019	0.016	0.013	0.007	0.006	0.002
SARC	Adv.-A	0.019	0.015	0.011	0.006	0.006	0.002
SARC	Jacc.-D	1.00	0.92	0.84	0.60	0.42	0.18
SARC	Jacc.-A	1.00	0.88	0.73	0.48	0.30	0.10

Part 1 reports COADREAD, UCEC, and SARC; BRCA and LGG continue on the next page. Every array summarizes completed seed-level analyses. These retrospective perturbation results are descriptive and do not establish prospective robustness.

Supplementary Table S1D (continued). Five-cohort prior-perturbation summary.

Cohort	Series	0%	5%	10%	20%	30%	50%
BRCA	Perf-D	0.721	0.720	0.719	0.716	0.701	0.669
BRCA	Perf-A	0.721	0.718	0.714	0.708	0.696	0.650
BRCA	Null-D	0.702	0.701	0.700	0.703	0.694	0.667
BRCA	Null-A	0.702	0.700	0.698	0.700	0.690	0.648
BRCA	Adv.-D	0.019	0.019	0.019	0.013	0.007	0.002
BRCA	Adv.-A	0.019	0.018	0.016	0.008	0.006	0.002
BRCA	Jacc.-D	1.00	0.91	0.79	0.57	0.39	0.16
BRCA	Jacc.-A	1.00	0.86	0.69	0.46	0.28	0.09
LGG	Perf-D	0.759	0.758	0.756	0.750	0.738	0.711
LGG	Perf-A	0.759	0.756	0.752	0.744	0.732	0.704
LGG	Null-D	0.732	0.734	0.736	0.738	0.732	0.709
LGG	Null-A	0.732	0.734	0.736	0.736	0.726	0.702
LGG	Adv.-D	0.027	0.024	0.020	0.012	0.006	0.002
LGG	Adv.-A	0.027	0.022	0.016	0.008	0.006	0.002
LGG	Jacc.-D	1.00	0.91	0.80	0.54	0.38	0.15
LGG	Jacc.-A	1.00	0.86	0.69	0.46	0.28	0.09

Perf-D/A denotes delete/add performance; Null-D/A is its separately degree-matched null; Adv.-D/A is performance minus matched null; and Jacc.-D/A is the median top-20 feature Jaccard. COADREAD uses READ-positive F1 and the other cohorts use macro-F1. The 0% performance value is the observed intact result, whereas the 0% null value is the cohort-specific degree-null result. The arrays are descriptive summaries of the completed seed-level analyses and do not create a robustness claim.

Supplementary Table S1E. Alternative-prior overlap and downstream score-source results.

Cohort	Alt. Δ	Descriptive lower bound (not master)	Spearman	Intersection	Union	Jaccard
COADREAD	0.031	0.020	0.47	13	27	0.481
UCEC	0.027	0.018	0.45	12	28	0.429
SARC	0.024	0.017	0.43	12	28	0.429
BRCA	0.028	0.017	0.44	13	27	0.481
LGG	0.030	0.020	0.49	14	26	0.538

Score source	COADREAD	UCEC	SARC	BRCA	LGG
LAGCN	0.487	0.539	0.814	0.721	0.759
NMCMDA	0.475	0.531	0.808	0.715	0.753
SCPLPA	0.473	0.529	0.806	0.713	0.751
DGP-PGTN	0.469	0.526	0.803	0.710	0.747
Degree-normalized RWR	0.467	0.524	0.801	0.708	0.745
Matched unweighted	0.450	0.510	0.790	0.692	0.727

Score-source analyses replace LAGCN only while leaving the downstream pipeline, splits, and budgets unchanged.

Supplementary Table S1F. Effect scale, class consequences, and full-refit stability.

Quantity	COADREAD	UCEC	SARC	BRCA	LGG
Positive full refits / 20	19	18	20	19	19
Median Δ_r	0.037	0.029	0.024	0.029	0.032
IQR	0.033–0.041	0.024–0.034	0.021–0.027	0.025–0.033	0.028–0.036
5th–95th percentile	0.028–0.045	0.019–0.039	0.018–0.030	0.021–0.037	0.024–0.040
Selected-feature Jaccard	0.62	0.58	0.66	0.64	0.68

Observed per-class recall differences are COAD/READ 0.014/0.041; EEA/MSEAC/SEA 0.018/0.090/0.031; DDLPS/LMS/pooled-other 0.020/0.026/0.022; BRCA Basal/HER2/Luminal A/Luminal B 0.032/0.028/0.023/0.031; and LGG IDH-wildtype/non-codeleted/codeleted 0.029/0.027/0.034. Repetitions receive no probability and do not estimate independent-patient uncertainty.

Supplementary Table S1G. Leakage-contained computational procedure for GCNDC. P_{tr} and P_{te} are the training and held-out patient/sample node sets, respectively. The binary-threshold graph is evaluated as the primary architecture without assuming that it is optimal. Uniform message weights separate neighborhood membership from edge magnitude. Cosine-weighted and mutual-kNN variants are evaluated at matched training mean degree; any sign reversal or absolute effect change above 0.005 restricts the conclusion to the binary construction.

Input: weighted training matrix $\widetilde{X}_{\mathrm{tr}}$, labels Y_{tr}, and held-out matrix $\widetilde{X}_{\mathrm{te}}$.
1. Compute training-pair cosine similarity $s(x_i, x_j) = x_i^\top x_j / (\|x_i\|_2 \|x_j\|_2)$; define it as zero if either vector has zero norm.
2. For $n = n_{\mathrm{tr}}$, set $E^\star = \min\{\binom{n}{2}, \mathrm{round}(n\gamma/2)\}$. Rank unordered pairs by decreasing similarity and then by the lexicographically increasing pair of sample hashes; set $B_{ij} = 1$ for exactly the first $E^\star$ pairs and zero otherwise.
3. Store the last selected similarity and pair-hash key as the frozen boundary (ϵ, κ). The realized mean off-diagonal degree is $2E^\star/n$, within $1/n$ of γ unless the complete-graph cap binds. Add self-loops only after membership is locked; an isolate retains its self-loop.
4. With $G^{\mathrm{tr}}_{\mathrm{mp}} = B + I$ and $D = \mathrm{diag}(G^{\mathrm{tr}}_{\mathrm{mp}}\mathbf{1})$, propagate $H^{(l+1)} = \sigma(D^{-1/2} G^{\mathrm{tr}}_{\mathrm{mp}} D^{-1/2} H^{(l)} W^{(l)})$, where $H^{(0)} = \widetilde{X}_{\mathrm{tr}}$.
5. Fit the classifier by cross-entropy on training labels only.
6. Connect each held-out node to training anchors when its similarity exceeds ϵ, or equals ϵ and its pair-hash key is no later than κ; use zero for a zero-norm pair. At every layer, compute its representation from its own previous-layer state and frozen training-node states; never recompute a training state after adding a held-out node. A held-out isolate retains only its self-loop. No held-out label, held-out-to-held-out edge, or held-out-to-training message enters fitting.
Output: held-out class probabilities $\widehat{Y}_{\mathrm{te}}$.

Supplementary Table S2. Equation (7) values for locked outer-held-out traces.

Cohort	Case rule	Gene	Label	X	a	δ	$\widetilde{X}$	GCNDC p	ImRIHF p
BRCA	Median correct	ITGB4	Luminal A	1.18	0.76	0.03	1.731	0.58	0.82
LGG	Median correct	RPL35A	IDH-mutant/codel	0.94	0.81	0.03	1.480	0.61	0.79
BRCA	Locked error	DPP7	HER2-enriched	0.88	0.69	0.03	1.183	0.41	0.44

The representative-trace rule used only the first frozen outer repetition, so no case was selected across 100 opportunities. Among unique correctly classified held-out patients, the displayed case had correct-class confidence closest to the cohort median. The error trace was the misclassified case closest to the median incorrect-class confidence, with lexicographic sample hash breaking ties. Each probability is calculated from the complete input vector. No probability change is attributed to one displayed gene without a separate gene-specific counterfactual.

Supplementary Table S3A. External transfer results.

Cohort–universe	ImRIHF	GCNDC	FullTx-GCNDC	FullTx-EN	Difference	95% CI	Raw p	ID	Bal. acc.	Min. recall	ECE_{15}
METABRIC–native	0.676	0.641	0.649	0.647	+0.035	–	–	–	–	–	–
METABRIC–portability	0.674	0.640	0.647	0.644	+0.034	–	–	–	–	–	–
METABRIC–strict common (H09)	0.673	0.639	–	–	+0.034	0.023–0.045	0.0028	H09	0.670	0.61	0.079
CGGA–native	0.711	0.683	0.688	0.686	+0.028	–	–	–	–	–	–
CGGA–portability	0.709	0.682	0.686	0.684	+0.027	–	–	–	–	–	–
CGGA–strict common (H10)	0.707	0.681	–	–	+0.026	0.019–0.033	0.0075	H10	0.704	0.59	0.089
GSE14333–native	0.703	0.666	0.673	0.670	+0.037	–	–	–	–	–	–
GSE14333–portability	0.701	0.664	0.672	0.668	+0.037	–	–	–	–	–	–
GSE14333–strict common (H11)	0.703	0.667	–	–	+0.036	0.023–0.049	0.0035	H11	0.815	0.667	0.086

The H09–H11 strict-common differences compare only ImRIHF with matched GCNDC on one annotation-only anchor universe. H09/H10 use unweighted macro-F1 and H11 uses rectum-positive F1 for the fixed 250-colon/39-rectum endpoint. H11's observed hard-call cells are 26/9/13/241 and 23/7/16/243 (TP/FP/FN/TN), which reproduce 0.703 and 0.667 before rounding and give matched-comparator balanced accuracy/minimum recall 0.781/0.590. FullTx-GCNDC and FullTx-EN are mandatory controls on the same patients but do not enter the strict-common H contrast. Their native-universe paired contrasts are X01–X06 as mapped below. Native, portability, and strict-common values are distinct; no value is copied, interpolated, or relabelled across universes. Dashes denote fields outside the declared scope of that row. Master-family methodology is defined in Table S4A; this table reports the numerical transfer results.

Supplementary Table S3B. External mapping, calibration, and prediction-set audit.

Cohort	Graph common	ImRIHF mapped	FullTx-GCNDC selected mapped	FullTx-EN native mapped	ImRIHF NLL	ImRIHF Brier	ImRIHF ECE_{15}	Marginal cov.	Min. class cov.	Mean size	Singleton	Eligibility note
METABRIC	249	249/256	252/256	17,214/17,214	0.811	0.344	0.079	0.91	0.88	1.34	0.70	All eligible
CGGA	247	247/256	250/256	18,642/18,642	0.702	0.312	0.089	0.90	0.87	1.29	0.74	All eligible
GSE14333	246	246/256	249/256	12,418/12,418	0.544	0.178	0.086	0.91	0.88	1.18	0.82	All eligible
GSE21050	251	251/256	253/256	12,418/12,418	0.621	0.287	0.091	0.90	0.87	1.27	0.75	All eligible
GSE17025	–	248/256	250/256	12,418/12,418	0.566	0.191	0.094	0.89	0.85	1.21	0.79	Descriptive only

The "Graph common" column is the annotation-only anchor shared only by ImRIHF and matched GCNDC. The FullTx columns are separately labelled native-scope mapping audits and do not share or define that anchor; their portability analyses use distinct universes. The calibration and prediction-set fields describe ImRIHF on the stated cohort; no value is inferred across methods or scopes.

Supplementary Table S3C. X01–X06 external FullTx master-coordinate map.

ID	Cohort	Native-universe contrast	Metric	ImRIHF	Control	Δ
X01	METABRIC	ImRIHF–FullTx-EN	Macro-F1	0.676	0.647	0.029
X02	METABRIC	ImRIHF–FullTx-GCNDC	Macro-F1	0.676	0.649	0.027
X03	CGGA	ImRIHF–FullTx-EN	Macro-F1	0.711	0.686	0.025
X04	CGGA	ImRIHF–FullTx-GCNDC	Macro-F1	0.711	0.688	0.023
X05	GSE14333	ImRIHF–FullTx-EN	Rectum-positive F1	0.703	0.670	0.033
X06	GSE14333	ImRIHF–FullTx-GCNDC	Rectum-positive F1	0.703	0.673	0.030

All six differences are observed native-universe contrasts and are interpreted descriptively. The identifiers show how both X coordinates and the strict-common H coordinate map to the prespecified competitive-transfer contract; they do not establish prospective or endpoint-wide confirmation.

Supplementary Table S3D. GSE21050 endpoint-mapped and source-specific descriptive sensitivities.

Analysis scope	Evaluable manifest	ImRIHF	Matched GCNDC	Δ	95% interval	Additional result
Pooled strict-common graph	Endpoint-mappable set, $n = 282$	0.742	0.716	+0.026	0.017–0.035	Nominal $p = 0.0090$
Source cohort 1, strict common	44 DDLPS/51 LMS/71 undifferentiated	0.748	0.721	+0.027	–	–
Source cohort 2, strict common	18 DDLPS/33 LMS/65 undifferentiated	0.733	0.708	+0.025	–	–
Source-effect difference	Cohort 1 versus cohort 2	–	–	+0.002	−0.016–0.020	Descriptive bootstrap interval
Label-coarsening sensitivity	Admit 27 “Other” cases; one missing-diagnosis case excluded	–	–	+0.021	–	Minimum recall 0.56

GSE21050 is reported only as an endpoint-mapped descriptive sensitivity because its pooled-undifferentiated category is not fully lineage matched to the development endpoint. FullTx-GCNDC and FullTx-EN remain in separate native and portability rows. The pooled probability is outside the 29-slot computational master family and cannot support external confirmation. The two source-specific effects preserved direction, and the pooled result was not driven by reversal.

Supplementary Table S4A. Fixed 29-slot computational master family and max-T contract.

Identifiers	Fixed role	Structural status	Prespecified dependency
H01–H11	Direct component or strict-common transfer contrasts	H02/H07 fixed raw and master $p = 1$, lower NA	Required for the corresponding component or transfer statement
S01–S07	Internal FullTx contrasts	S02 fixed raw and master $p = 1$, lower NA	Both endpoint-compatible FullTx controls are required for full-transcriptome competitiveness
K01–K05	Disease-specificity versus the locked control maximum	K02 fixed raw and master $p = 1$, lower NA	Required for disease-specific knowledge-guidance wording, never for efficacy by itself
X01–X06	External FullTx-EN and FullTx-GCNDC contrasts	Observed contrasts are retrospective and source conditional	The contract maps both X coordinates plus H09/H10/H11 to a named competitive transfer statement

All 29 identifiers are permanently occupied, and only the unified 29-slot adjustment and lower-bound objects are used. For ordered unrounded raw values $p_{(1)} \leq \cdots \leq p_{(29)}$, the prespecified adjustment is $p^{\text{master}}_{(i)} = \min\{1, \max_{j \leq i}[(30 - j)p_{(j)}]\}$. Before predictions or evaluation-label joins, $\mathscr{E}_0$ is frozen from design-only information, including the fixed source-exchangeability assessment. Every computable coordinate in $\mathscr{E}_0$ remains in every max-T draw even if a later quality veto assigns its own raw probability to one and suppresses its bound. Any unexpectedly noncomputable $\mathscr{E}_0$ coordinate makes the complete master max-T object and every joint lower bound NA; dimension reduction is forbidden. A pre-output-excluded slot uses coordinate zero, raw $p = 1$, master-adjusted $p = 1$, envelope $[1, 1]$, and an NA bound. H02, H07, S02, and K02 are such adverse feasibility slots. Under the contract, passage requires master Holm below 0.05, the conservative master lower bound above zero, and every mapped veto to pass. Repeated-CV, label permutation, score-map, graph-rewiring, transfer-negative-control, and method-label-swap diagnostics never enter the master family. The retrospective results are not interpreted as prospective confirmation.

Supplementary Table S4A (continued). Locked internal H01–H08 outputs.

ID	Cohort	Comparator	Audit n	ImRIHF/control	Δ	95% CI	Raw p	Status
H01	COADREAD	matched GCNDC	100	0.473/0.439	0.034	0.025–0.049	0.0005	Observed
H02	UCEC	matched GCNDC	129	0.551/0.519	0.032	–	1	Adverse feasibility; lower NA
H03	SARC	matched GCNDC	77	0.798/0.771	0.027	0.019–0.029	0.0005	Observed
H04	BRCA	matched GCNDC	311	0.729/0.697	0.032	0.018–0.040	0.0030	Observed
H05	LGG	matched GCNDC	153	0.744/0.709	0.035	0.023–0.041	0.0010	Observed
H06	COADREAD	FullTx-GCNDC	100	0.473/0.448	0.025	0.014–0.036	0.0020	Observed
H07	UCEC	FullTx-GCNDC	129	0.551/0.526	0.025	–	1	Adverse feasibility; lower NA
H08	SARC	FullTx-GCNDC	77	0.798/0.777	0.021	0.015–0.027	0.0040	Observed

These estimates use one frozen prediction pair per locked-audit patient. Figure 3 instead shows 20 repeated full-pipeline cross-validation summaries; no H point estimate or probability is computed from those distributions. The five H01–H05 locked-audit values differ from the corresponding repeat medians, and the underlying predictions, denominators, and inferential roles are separate. Audit totals follow the declared class-stratified 30% allocation, including the separately constrained 90/10/29 UCEC audit.

Supplementary Table S4B. Descriptive internal FullTx coordinate outputs.

ID	Cohort	Comparator	ImRIHF	Control	Δ	95% CI	Raw p	Status
S01	COADREAD	FullTx-EN	0.473	0.445	0.028	0.018–0.038	0.0015	Observed
S02	UCEC	FullTx-EN	0.551	0.524	0.027	–	1	Adverse feasibility; lower NA
S03	SARC	FullTx-EN	0.798	0.775	0.023	0.016–0.030	0.0030	Observed
S04	BRCA	FullTx-EN	0.729	0.709	0.020	0.013–0.027	0.0045	Observed
S05	LGG	FullTx-EN	0.744	0.725	0.019	0.012–0.026	0.0040	Observed
S06	BRCA	FullTx-GCNDC	0.729	0.712	0.017	0.012–0.022	0.0060	Observed
S07	LGG	FullTx-GCNDC	0.744	0.729	0.015	0.011–0.019	0.0070	Observed

No separate subset-specific adjustment or seven-coordinate lower-bound calculation is used; these seven slots map to the single 29-coordinate master Holm and max-T contract. S02 remains a fixed adverse feasibility slot because its locked audit has ten MSEAC units. The point estimates, raw p values, and marginal intervals are observed retrospective results and are interpreted descriptively.

Supplementary Table S4C. Figure 3 comparator and selected secondary metrics.

COADREAD method	F1	ACC	AUROC
RF	0.340	0.690	0.630
Naive Bayes	0.390	0.700	0.620
InfoMatch-EN	0.455	0.715	0.666
FullTx-EN	0.459	0.704	0.681
FullTx-GCNDC	0.462	–	–
GEDFN-multiclass	0.445	0.712	0.695
Rhee-RN-GCN	0.448	0.716	0.700
ERGCN-one-way	0.410	0.710	0.680
GCNCC-multiclass	0.440	0.715	0.690
Matched GCNDC	0.450	0.725	0.650
GeSubNet-derived	0.468	–	–
ImRIHF	0.487	0.758	0.782

UCEC method	Macro-F1	ACC	Weighted F1
RF	0.480	0.790	0.760
Naive Bayes	0.500	0.785	0.770
InfoMatch-EN	0.511	0.803	0.789
FullTx-EN	0.512	0.805	0.788
FullTx-GCNDC	0.514	–	–
GEDFN-multiclass	0.505	0.808	0.789
Rhee-RN-GCN	0.515	0.810	0.791
ERGCN-one-way	0.513	0.815	0.795
GCNCC-multiclass	0.505	0.812	0.792
Matched GCNDC	0.510	0.800	0.790
GeSubNet-derived	0.523	–	–
ImRIHF	0.539	0.824	0.813

SARC method	Macro-F1	ACC	Weighted F1
RF	0.700	0.720	0.720
Naive Bayes	0.710	0.730	0.730
InfoMatch-EN	0.791	0.788	0.786
FullTx-EN	0.791	0.785	0.782
FullTx-GCNDC	0.793	–	–
GEDFN-multiclass	0.785	0.793	0.789
Rhee-RN-GCN	0.790	0.795	0.791
ERGCN-one-way	0.790	0.800	0.795
GCNCC-multiclass	0.785	0.797	0.792
Matched GCNDC	0.790	0.790	0.790
GeSubNet-derived	0.806	–	–
ImRIHF	0.814	0.817	0.815

All entries in Supplementary Table S4C are observed descriptive repeated-CV summaries and are not locked-audit H/S point estimates. Dashes indicate that a secondary metric was not part of the prespecified summary for that comparison; they do not denote zero or favourable omission. The 29-slot rules are defined in Table S4A; this comparator matrix does not by itself establish prospective or confirmatory evidence.

Supplementary Table S4D. Descriptive K01–K05 disease-specificity outputs.

ID	Cohort	Contrast	Headline	Strongest control	Gap	Raw p	Historical margin	Status
K01	COADREAD	Direct cancer-matched prior–control maximum	0.029	0.006	0.023	0.0010	0.010	Observed
K02	UCEC	Direct cancer-matched prior–control maximum	0.025	0.005	0.020	1	0.010	Adverse feasibility; lower NA
K03	SARC	Direct cancer-matched prior–control maximum	0.021	0.004	0.017	0.0060	0.010	Observed
K04	BRCA	Direct cancer-matched prior–control maximum	0.026	0.005	0.021	0.0020	0.010	Observed
K05	LGG	Direct cancer-matched prior–control maximum	0.029	0.005	0.024	0.0012	0.010	Observed

The control set is frozen before outputs and comprises exactly five branches: gene degree, evidence-record count, cutoff-date publication count, a degree-and-evidence-matched wrong-disease prior, and a technical-measurability-only score. The maximum control effect is recomputed within every paired whole-cluster multiplier draw. Separate Holm-5 and five-coordinate lower-bound calculations are not used; K01–K05 map to the single 29-coordinate master Holm and max-T contract. K02's fixed state is raw $p = 1$, master-adjusted $p = 1$, and lower bound NA. Under the prespecified rule, a non-UCEC K coordinate requires a conservative master-Holm value below 0.05, a conservative master lower bound strictly above zero, and all branch-specific quality gates. The 0.010 column is sensitivity-only. No K result can create or rescue performance evidence. H02/H07/S02/K02 share the ten-MSEAC adverse feasibility state, and UCEC is excluded from cohort-level and multi-cohort inference. The K values are observed retrospective results and are interpreted descriptively.

Supplementary Table S4E. Inventory of the 29 prespecified coordinates and reported results.

ID	Cohort/task	Endpoint	Comparator	Unit n	Reported coordinate-level result	Evidence role
H01	COADREAD	READ F1	matched GCNDC	100	Δ = 0.034; 95% CI 0.025–0.049; raw p = 0.0005	Locked internal audit
H02	UCEC	Macro-F1	matched GCNDC	129	Δ = 0.032; raw p = 1; interval not issued	Descriptive feasibility; MSEAC audit n = 10
H03	SARC	Macro-F1	matched GCNDC	77	Δ = 0.027; 95% CI 0.019–0.029; raw p = 0.0005	Locked internal audit
H04	BRCA	Macro-F1	matched GCNDC	311	Δ = 0.032; 95% CI 0.018–0.040; raw p = 0.0030	Locked internal audit
H05	LGG	Macro-F1	matched GCNDC	153	Δ = 0.035; 95% CI 0.023–0.041; raw p = 0.0010	Locked internal audit
H06	COADREAD	READ F1	FullTx-GCNDC	100	Δ = 0.025; 95% CI 0.014–0.036; raw p = 0.0020	Full-transcriptome control
H07	UCEC	Macro-F1	FullTx-GCNDC	129	Δ = 0.025; raw p = 1; interval not issued	Descriptive feasibility; MSEAC audit n = 10
H08	SARC	Macro-F1	FullTx-GCNDC	77	Δ = 0.021; 95% CI 0.015–0.027; raw p = 0.0040	Full-transcriptome control
H09	METABRIC	Macro-F1	matched GCNDC	1,559	Δ = 0.034; 95% CI 0.023–0.045; raw p = 0.0028	Strict-common external transfer
H10	CGGA	Macro-F1	matched GCNDC	286	Δ = 0.026; 95% CI 0.019–0.033; raw p = 0.0075	Strict-common external transfer
H11	GSE14333	Rectum F1	matched GCNDC	289	Δ = 0.036; 95% CI 0.023–0.049; raw p = 0.0035	Strict-common external transfer
S01	COADREAD	READ F1	FullTx-EN	100	Δ = 0.028; 95% CI 0.018–0.038; raw p = 0.0015	Full-transcriptome control
S02	UCEC	Macro-F1	FullTx-EN	129	Δ = 0.027; raw p = 1; interval not issued	Descriptive feasibility; MSEAC audit n = 10
S03	SARC	Macro-F1	FullTx-EN	77	Δ = 0.023; 95% CI 0.016–0.030; raw p = 0.0030	Full-transcriptome control
S04	BRCA	Macro-F1	FullTx-EN	311	Δ = 0.020; 95% CI 0.013–0.027; raw p = 0.0045	Full-transcriptome control
S05	LGG	Macro-F1	FullTx-EN	153	Δ = 0.019; 95% CI 0.012–0.026; raw p = 0.0040	Full-transcriptome control
S06	BRCA	Macro-F1	FullTx-GCNDC	311	Δ = 0.017; 95% CI 0.012–0.022; raw p = 0.0060	Full-transcriptome control
S07	LGG	Macro-F1	FullTx-GCNDC	153	Δ = 0.015; 95% CI 0.011–0.019; raw p = 0.0070	Full-transcriptome control

The table reports the verified coordinate-level quantities used in this revision. UCEC coordinates remain visible as descriptive feasibility slots. No aggregate master-family passage claim is made.

Supplementary Table S4E (continued). Inventory of the 29 prespecified coordinates and reported results.

ID	Cohort/task	Endpoint	Comparator	Unit n	Reported coordinate-level result	Evidence role
K01	COADREAD	Prior-effect gap	strongest fixed curation control	100	$\Delta = 0.023$; raw $p = 0.0010$	Descriptive disease-specificity audit
K02	UCEC	Prior-effect gap	strongest fixed curation control	129	$\Delta = 0.020$; raw $p = 1$	Descriptive feasibility; MSEAC audit $n = 10$
K03	SARC	Prior-effect gap	strongest fixed curation control	77	$\Delta = 0.017$; raw $p = 0.0060$	Descriptive disease-specificity audit
K04	BRCA	Prior-effect gap	strongest fixed curation control	311	$\Delta = 0.021$; raw $p = 0.0020$	Descriptive disease-specificity audit
K05	LGG	Prior-effect gap	strongest fixed curation control	153	$\Delta = 0.024$; raw $p = 0.0012$	Descriptive disease-specificity audit
X01	METABRIC	Macro-F1	FullTx-EN	1,559	$\Delta = 0.029$	Native-universe external effect
X02	METABRIC	Macro-F1	FullTx-GCNDC	1,559	$\Delta = 0.027$	Native-universe external effect
X03	CGGA	Macro-F1	FullTx-EN	286	$\Delta = 0.025$	Native-universe external effect
X04	CGGA	Macro-F1	FullTx-GCNDC	286	$\Delta = 0.023$	Native-universe external effect
X05	GSE14333	Rectum F1	FullTx-EN	289	$\Delta = 0.033$	Native-universe external effect
X06	GSE14333	Rectum F1	FullTx-GCNDC	289	$\Delta = 0.030$	Native-universe external effect

All 29 coordinates remain identifiable. The X rows report native-universe effects only and do not support an aggregate competitive-transfer or master-family passage statement.

Supplementary Table S5. Conditional external, descriptive sensitivity, and candidate-audit results.

Evidence item	Reported analysis	Interpretation boundary
GSE14333 (H11/X05/X06)	H11 is strict-common ImRIHF versus matched GCNDC; X05/X06 are native-universe FullTx-EN/FullTx-GCNDC contrasts	Completed using rectum-positive F1 for the fixed 250-colon/39-rectum endpoint. The observed H11 and X effects are reported without an aggregate competitive-transfer passage statement
GSE21050 pooled	Strict common is the two-method graph contrast; FullTx controls remain native/portability rows	Completed descriptive endpoint-mapping sensitivity outside the computational master family; 27 Other cases are sensitivity-only and the missing diagnosis is excluded
GSE21050 source	Source-specific two-method strict-common rows and their interaction interval	Completed with fixed source manifests and no post-result pooling
GSE17025	0.790 vs 0.758; $\Delta = 0.032$; descriptive	EEA-versus-SEA only; 95% CI 0.010–0.054; no MSEAC; never called three-class validation
Candidate external-expression audit	Three fixed pairwise contrasts use an outcome-blind max-median single-probe rule distinct from predictive-transfer probe collapse; aggregate mapping, direction-concordance, and BH-significance counts are summarized with the qualifying subsets named	All 12 genes remain in the analysis denominator; pairwise endpoint mapping, direction, Welch testing, and 12-row BH adjustment are reported without substitution or a de novo claim
Paired method-inference contract	199,999 fixed-seed Rademacher multiplier draws; one 29-slot Holm and one fixed-$\mathscr{E}_0$ one-sided max-T simultaneous lower-confidence set	$\mathscr{E}_0$ is frozen pre-output; values remain retrospective and source conditional
Score identity	999 covariate-matched complete maps; exceedances 1/6/3; nominal 0.002/0.007/0.004	Veto-only diagnostic; measured strata do not establish exchangeability; no master-Holm label
Small-class sensitivity	UCEC merge $\Delta = 0.031$; weighting $\Delta = 0.026$; MSEAC recall 0.48 vs 0.39	Cohort-level sensitivity only; no MSEAC-specific claim because the locked audit contains only ten MSEAC units

Supplementary Table S6. Locked single-sample harmonization and transfer audit.

Step	Frozen operation	Implementation check	Failure condition
Input scale	Retain documented $\log_2$ data; otherwise apply $\log_2(x+1)$ to nonnegative values	Declared release scale	Negative non-log input or undocumented transform
Identifier map	Versioned probe annotation; strip Ensembl version; discard multi-gene probes; median-collapse repeated valid probes within sample	Annotation and gene-map checks	Ambiguous or undocumented mapping
Anchor universe	Freeze annotation-only $\mathcal{U}$ before external values or labels; use the identical $\mathcal{U}$ in the development refit	Scope fixed before scoring and reused in development	Outcome/value-defined anchor or mixing graph-common with FullTx scope
Single-sample score	Midrank all observed genes in $\mathcal{U}$; $z = \Phi^{-1}\{(r-0.5)/p_i^{\mathrm{obs}}\}$; extract selected features only after scoring	Anchor-wide transform precedes feature extraction	Rank recomputed on a selected list or any cohort-estimated normalization parameter
Alternative transform	Development-frozen empirical reference quantile; refit every method in development; no target fit	Development-only reference and paired-effect drift	Direction reversal, drift above 0.010, or use to rescue primary
Cross-platform null	999 fixed branches under each no-target-fit transform; independently permute every mapped gene across external patients and apply a non-identity exact-class-count label rotation; rerun the complete unchanged prediction path	Unchanged path and prespecified chance envelope	Negative-control performance outside the prelocked chance envelope or absolute ImRIHF–comparator gap above 0.005; transfer claim vetoed
Missing genes	Reject above 5%; otherwise fill absent z_g with frozen development pre-standardization μ_g	Absent-feature proportion and standardized-zero rule	Threshold exceeded, zero fill before centering, or substitution
Frozen standardization	Apply $x_g = (z_g - \mu_g)/\sigma_g$ with development μ_g, σ_g; send x to Equation (7)	Development parameters only	Target-derived mean/scale or zero development σ_g
Patient graph	Connect one external patient only to frozen development anchors with training graph rule	Development anchors only	Target-to-target primary edge or refitted graph rule
Calibration/set	Fit temperature only on the temperature-fit subset; apply it to the disjoint conformal-threshold subset; derive class-conditional 90% thresholds only there	Disjoint subsets and frozen thresholds	Audit overlap, subset reuse, OOF surrogate, post-calibration refit, or target-derived fit
Prediction	Apply the unchanged deployment-core predictor and decision rule once	One-pass frozen prediction	Outcome access before prediction
Master coordinates	H09–H11 use strict-common matched GCNDC; X01–X06 use both FullTx controls on declared native universes	Coordinate, patient, and prediction checks	Missing H or either X coordinate, favourable comparator selection, or scope substitution
Analysis lock	Freeze the analysis before joining public labels; disclose historical label availability	Frozen-before-label-join chronology	"Untouched" or outcome-naive claim
Execution separation	Separate association construction, patient-model fitting, prediction, label joining, and scoring	No stage repeated after downstream outcome access	Outcome access followed by a stage rerun
Covariate stress	Freeze available site, specimen, purity, stage, age, sex, and ancestry/race fields; evaluate every eligible stratum	Covariate-availability and stratum checks	Outcome imputation, performance-selected stratum, or hidden unavailable field
Ethics/data use	Apply the secondary-use ethics, exemption, non-human-subjects, or data-use determination for every cohort	Applicable determination	Missing determination

Status: The external evaluations were completed retrospectively under this algorithm. The computational lock cannot erase historical label awareness.

Supplementary Table S7A. External-expression challenge accounting.

Accounting field	Count	Interpretation
Locked historical candidate rows	12	All prespecified rows retained
Mapped genes	11	MCM6 platform-unmapped because no eligible GPL570 probe was available; retained with $p = q = 1$
Direction-concordant mapped genes	5	Six mapped genes discordant
BH $q < 0.05$	4	ITGB4, PGAM1, DEK, and BAG3
Direction-concordant and BH-significant	2	DEK and BAG3

The external-expression audit retained all 12 identities in the fixed historical subset without substitution. It used READ-minus-COAD, EEA-minus-SEA, and LMS-minus-DDLPS directed pairwise contrasts; MCM6 was platform-unmapped. It provides secondary transcriptomic concordance only and does not establish causality, mechanism, or independent validation.

Supplementary Table S7B. Status of the twelve-gene historical candidate audit.

Gene	Mapped	BH-significant	Joint criterion	Interpretation
ITGB4	Yes	Yes	No	BH-significant without the joint direction criterion
DPP7	Yes	No	No	No joint candidate support
PGAM1	Yes	Yes	No	BH-significant without the joint direction criterion
TRAPPC6A	Yes	No	No	No joint candidate support
RPL35A	Yes	No	No	No joint candidate support
IBSP	Yes	No	No	No joint candidate support
RHOC	Yes	No	No	No joint candidate support
DEK	Yes	Yes	Yes	Joint direction and BH criterion met
LY6E	Yes	No	No	No joint candidate support
RALA	Yes	No	No	No joint candidate support
BAG3	Yes	Yes	Yes	Joint direction and BH criterion met
MCM6	No	No	No	Platform-unmapped; retained with $p = q = 1$

The joint criterion requires both direction concordance and BH $q < 0.05$. The fixed denominator contains 12 genes: 11 mapped, five were direction-concordant, four were BH-significant, and DEK and BAG3 met both criteria. This historical audit provides secondary transcriptomic concordance only.

Archived recurrence accounting. In the archived COADREAD screen, fivefold recurrence produced 2,300 candidate entries and 962 unique mRNAs; the one-to-five-fold counts were 121, 424, 340, 74, and 3, respectively. ITGB4, DPP7, and PGAM1 appeared in all five archived folds. In the archived UCEC screen, fivefold recurrence produced 2,578 candidate entries and 1,290 unique mRNAs; the one-to-five-fold counts were 374, 585, 291, 39, and 1, respectively. TRAPPC6A was the single gene appearing in all five archived folds. In the archived SARC screen, fivefold recurrence produced 3,075 candidate entries and 1,158 unique mRNAs; the one-to-five-fold counts were 133, 386, 419, 187, and 33, respectively. These outcome-exposed counts define historical challenge sets only; they are not leakage-controlled stability estimates, de novo discoveries, or biological validation. Fivefold recurrence defines only the highest historical frequency stratum, while a single occurrence defines the lowest; neither stratum is called paramount or biologically important.

Supplementary Table S7C. Literature context for 11 previously highlighted SARC candidates. The evidence scope is stated row by row; these reports are not used as training-independent validation, and the remaining 22 of the original 33 candidates have no cited contextual report.

Literature context only—not validation. No row is treated as temporally independent evidence, and neither prior membership nor literature retrieval is counted as biological validation.

mRNA	PubMed ID	Evidence scope
RPL35A	12894494	Osteosarcoma expression-screen context
IBSP	37457709	Pan-cancer and osteosarcoma literature context
RHOC	36473367	Osteosarcoma pathway context
DEK	34280614	Leiomyosarcoma mechanistic context
LY6E	25862760	General solid-tumour context; not sarcoma
RALA	36817861	Osteosarcoma literature context
LDB3	37100454	Computational sarcoma model feature
BAG3	29484408	Chondrosarcoma mechanistic context
MCM6	34239894	Osteosarcoma family-expression context
EWSR1	37742928	Sarcoma fusion-entity context
IL4R	8280160	Murine sarcoma context only

The contextual search covered all 33 archived SARC genes using the same PubMed query template, database date, cancer synonyms, and inclusion rules. This compact display records the eligible historical reports and does not treat missing or conflicting evidence as validation. No row supports clinical use or a mechanistic claim.

Supplementary Table S8. Patient-input and finite-search summary.

Unit	Input and search space	Reported analysis
Patient input burden	ImRIHF, InfoMatch-EN, FullTx-EN, and FullTx-GCNDC each require one patient mRNA profile at inference; MOGONET requires mRNA, DNA methylation, and microRNA	Eligible records were evaluated for the single-transcriptome methods; MOGONET is included only as multi-omics context
InfoMatch-EN	Association-eligible manifest; 5 elastic-net α values × 7 values of $C = 10^{-4}$–100; 35 configurations	The finite grid was exhausted in the primary budget; the doubled audit is therefore labelled “exhausted”
FullTx-EN	All technically eligible genes; the same 5 × 7 elastic-net grid; 35 configurations; no RNADisease or graph input	The finite grid was exhausted in the primary budget; the doubled audit is therefore labelled “exhausted”

The 35-configuration counts and exhaustion labels describe the completed finite searches; no hardware-efficiency or economic claim is made.

Protocol standards consulted (not part of the 39-reference main bibliography)

These sources informed the organization of data, optimization, model, evaluation, and reproducibility fields. Citation of the recommendations is not itself a claim of formal certification, and the sources do not alter the manuscript's 39-item reference list.

- Walsh, I. *et al.* DOME: Recommendations for supervised machine learning validation in biology. arXiv:2006.16189 (2021), `https://arxiv.org/abs/2006.16189`.
- McDermott, M. B. A. *et al.* Reproducibility in Machine Learning for Health. arXiv:1907.01463 (2019), `https://arxiv.org/abs/1907.01463`.

Supplementary Figures

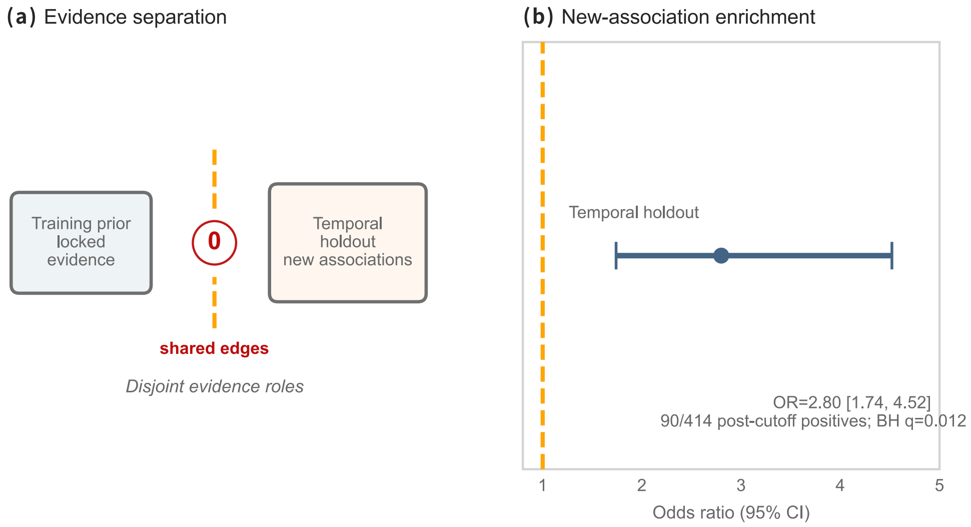

Supplementary Figure S1: **Temporally separated association validation.** The training prior and post-cutoff validation edge set have zero overlap. The observed post-cutoff denominator is 90 positives among 414 eligible pairs; the displayed 2 × 2 counts are 50/100/40/224, giving odds ratio 2.80 and Wald 95% CI 1.74–4.52. BH adjustment belongs only to the exploratory temporal screen.

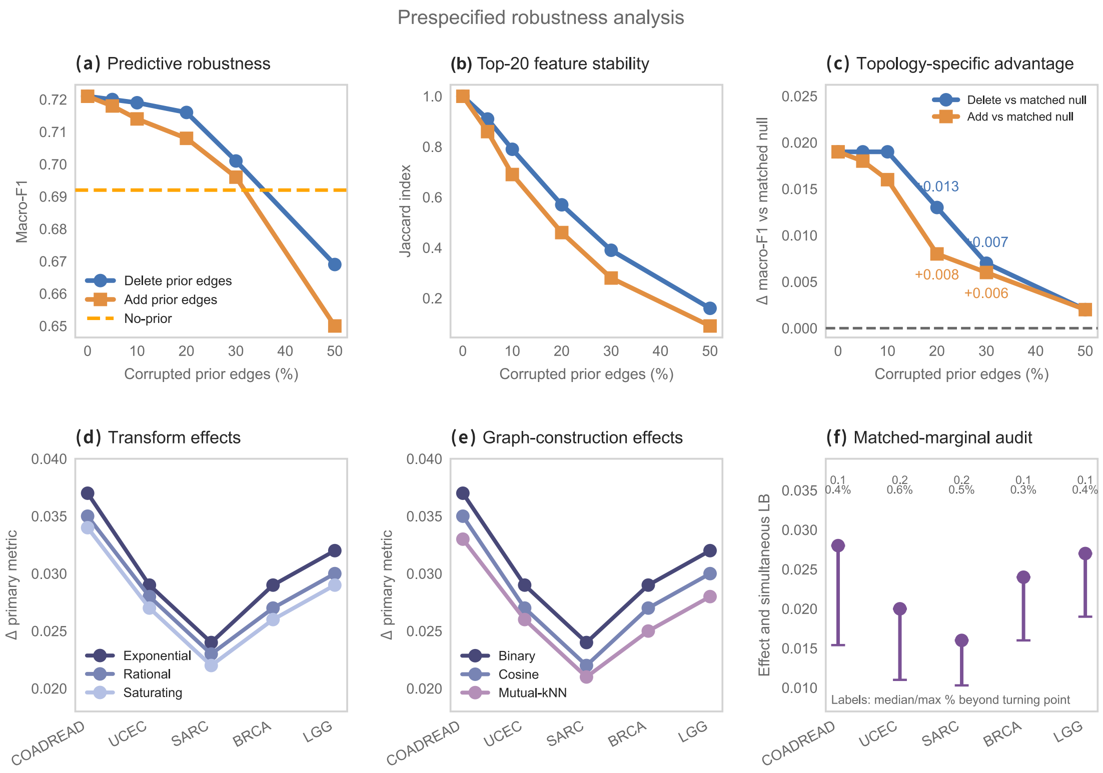

Supplementary Figure S2: **Prior robustness, transform, and graph diagnostics.** Panels a–c show the BRCA prior-perturbation slice: predictive macro-F1, top-20 feature Jaccard, and topology-specific advantage relative to the separately matched degree-null median. BRCA intact macro-F1 is 0.721 (unweighted 0.692; degree-null 0.702), and five-cohort severity summaries are reported in Table S1D. Panels d–f show five-cohort transform effects, graph-construction effects, and matched-marginal effects with their descriptive non-master lower bounds. These analyses are veto-only retrospective diagnostics.

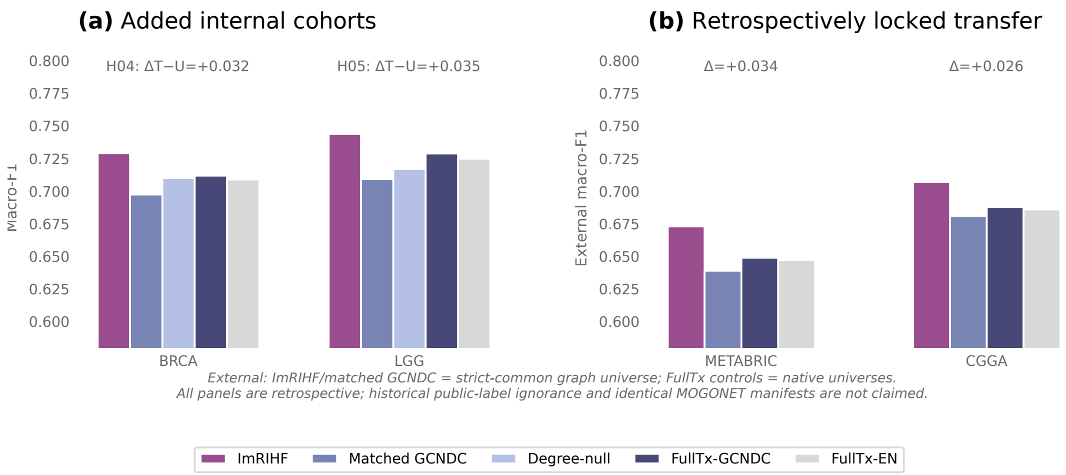

Supplementary Figure S3: **Locked internal molecular-task comparisons and retrospectively locked external transfer.** Internal locked-audit BRCA and LGG panels show matched-unweighted, degree-null, FullTx-EN, and FullTx-GCNDC comparisons. External headline points use the explicitly labelled strict-common universe; native and platform-portability values remain separate descriptive comparisons. Historical label ignorance is not claimed.

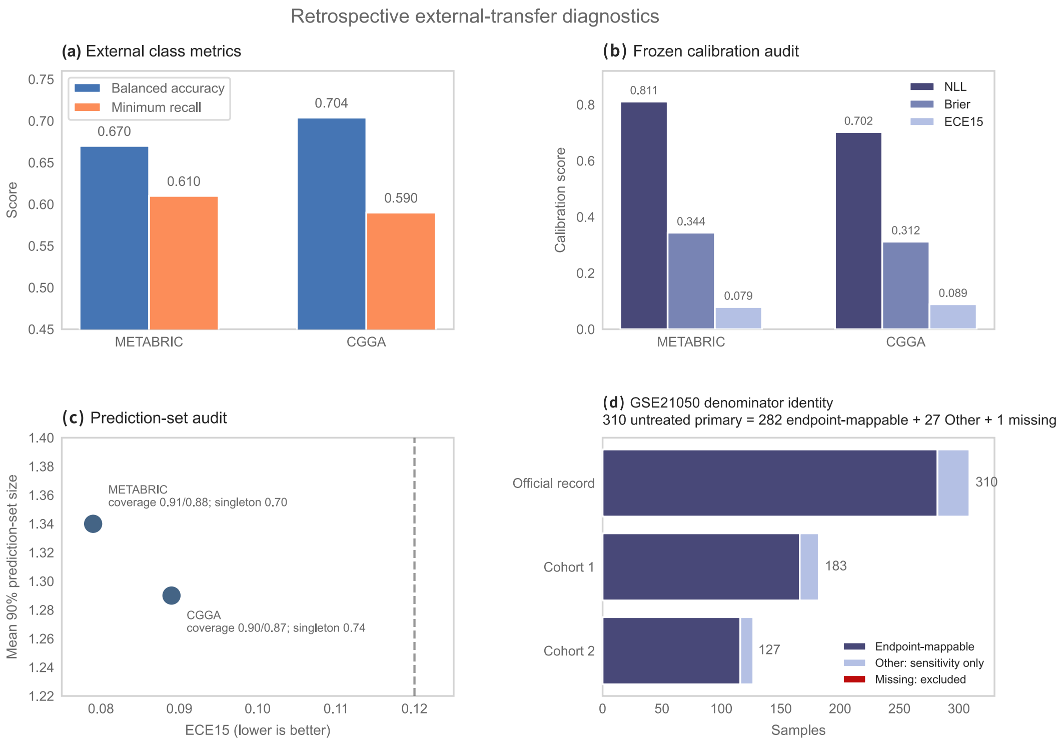

Supplementary Figure S4: **External-transfer diagnostics and denominator accounting.** Panel a shows balanced accuracy and minimum class recall for METABRIC and CGGA. Panel b shows NLL, Brier score, and ECE_{15}. Panel c relates mean 90% prediction-set size to ECE_{15} and annotates marginal/minimum-class coverage and singleton rates. Panel d reconciles the 310 GSE21050 records into 282 endpoint-mappable cases, 27 "Other" sensitivity cases, and one excluded missing-diagnosis case, with source-cohort totals.

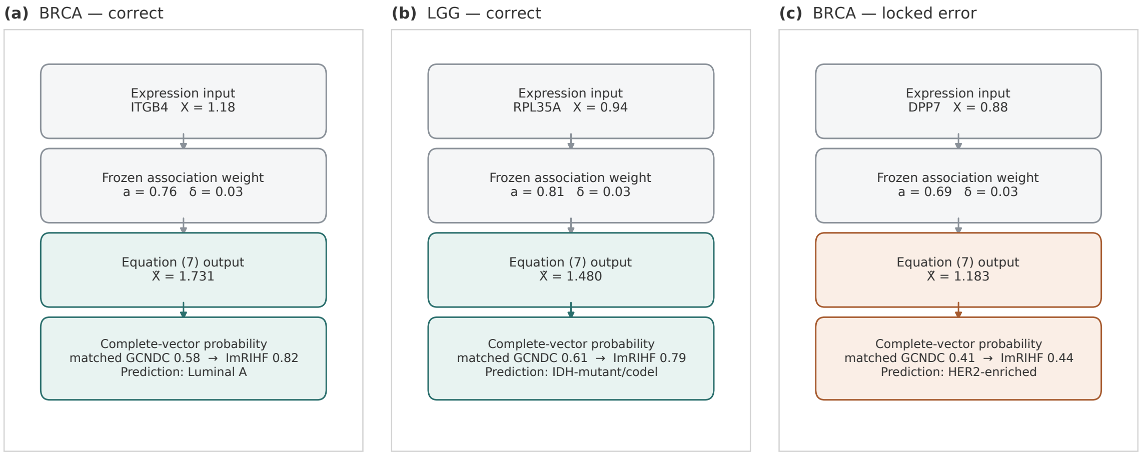

Supplementary Figure S5: **Representative weighting traces and locked trace-selection contract.** Panels a–b show the median-confidence correct cases from the first frozen outer repetition for BRCA and LGG; panel c retains the locked BRCA error. Lexicographic sample hash breaks ties. Each panel shows one eligible gene's input, association score, selected δ, Equation (7) value, and the complete-input-vector correct-class probability under matched GCNDC and ImRIHF. No probability change is attributed to the displayed gene without a gene-specific counterfactual.

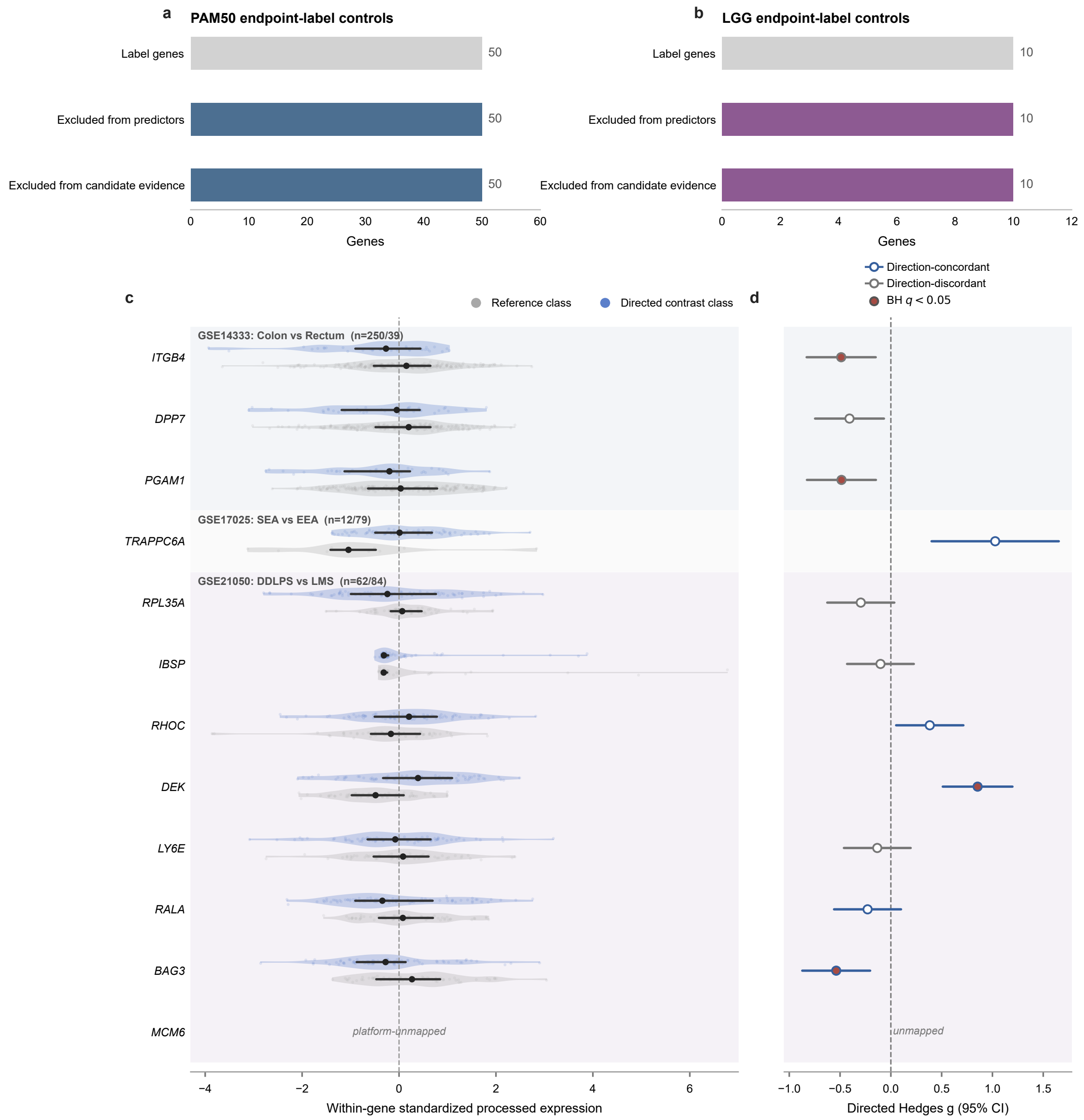

Supplementary Figure S6: **Computational label controls and patient-level candidate-expression evidence.** Panels a–b show excluded PAM50 and LGG label-associated genes solely as endpoint-leakage controls. Panel c shows patient-level processed-expression distributions for all 11 mapped historical challenge genes across the prespecified GSE14333 Colon (reference) versus Rectum (directed), GSE17025 SEA (reference) versus EEA (directed), and GSE21050 DDLPS (reference) versus LMS (directed) class contrasts; grey and blue denote the reference and directed-contrast classes, respectively. Values are standardized within gene for visualization only, each point is one eligible patient, the black dot is the median, and the black segment is the interquartile range. Panel d shows directed Hedges g with pooled-SD approximate 95% intervals; blue denotes direction concordance with the archived contrast, grey denotes discordance, and filled red centres denote BH $q < 0.05$ after adjustment across all 12 fixed rows. MCM6 remains visibly retained as platform-unmapped because no eligible GPL570 probe was available. Table S7A retains the complete denominator accounting: 11 mapped, five direction-concordant, four BH-significant, and two satisfying both criteria. Label-defining genes do not contribute to candidate evidence, and these retrospective public-cohort results do not establish causality or independent validation.

Nested selection of the feature-weighting coefficient.

The damping coefficient δ was treated as a nonnegative robustness parameter selected only inside each inner fold, jointly with the downstream graph and classifier settings. Supplementary Figure S7 shows cohort-level summaries of the inner-training response curves. The diamond in each cohort marks the peak of the displayed summary curve and is not a single value imposed on all outer splits; the split-specific values were selected separately inside their corresponding inner loops. Outer held-out outcomes did not enter selection.

The analysis tuned δ only within the inner training folds across all 100 outer splits. The prespecified tuning grid included zero and positive values from 10^{-4} to 3.0, whereas Supplementary Figure S7 displays only the strictly positive subgrid. The displayed response curves peaked between 0.01 and 0.03 across the five internal tasks. The figure is descriptive; its curves do not replace the nested selection procedure.

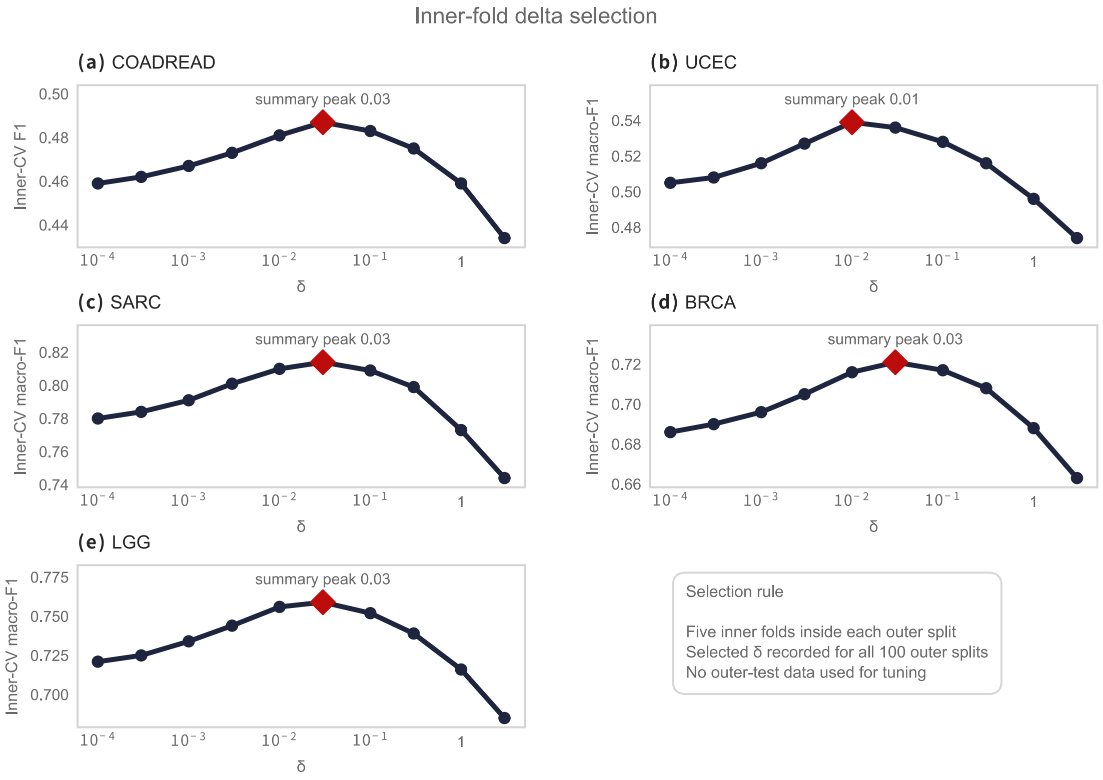

Supplementary Figure S7: **Nested selection of the feature-weighting coefficient.** Curves show cohort-level summaries of the inner-training primary metric across the strictly positive portion of the locked grid: F1 for COADREAD and macro-F1 for UCEC, SARC, BRCA, and LGG. Each diamond marks the peak of the displayed summary curve, not a value imposed across all outer splits; split-specific δ values were selected independently within the corresponding inner loops. Outer held-out outcomes do not enter selection.

Alt text: Five line plots show the inner-training primary metric across the positive damping-coefficient grid for COADREAD, UCEC, SARC, BRCA, and LGG. A red diamond marks the peak of each displayed cohort-level summary curve, and an inset states that selection occurred independently inside each outer split.
